# Distinctive properties of the prion protein in the brain and retina in the amyloidosis associated with the PRNP F198S Mutation *

**DOI:** 10.21203/rs.3.rs-9441514/v1

**Published:** 2026-07-16

**Authors:** Bernardino Ghetti, Bradley S. Glazier, Michele Fiorini, Kathy L. Newell, José M. Bonnin, Jill R. Murrell, Leah Rie Varner, Max Jacobsen, James F. Striebel, Suzette A. Priola, Gianluigi Zanusso

**Affiliations:** 1 Department of Pathology & Laboratory Medicine, Indiana University School of Medicine, 635 Barnhill Drive MS A138, Indianapolis, Indiana 46202, USA; 2 Department of Neurosciences, Biomedicine and Movement Sciences, University of Verona, L.A. Scuro, 10, Verona, 37134, Italy; 3 Rocky Mountain Laboratories, Laboratory of Neurological Infections and Immunity, National Institute of Allergy & Infectious Diseases, National Institutes of Health, Hamilton, MT USA

**Keywords:** Gerstmann-Sträussler-Scheinker disease, retina, prion, *PRNP* gene, *PRNP* F198S mutation

## Abstract

Prion protein (PrP) deposits in the form of diffuse and cored plaques occur in most gray matter areas of the brain of individuals affected by Gerstmann-Strässler-Scheinker disease (GSS) associated with the F198S mutation in *PRNP*; however, the PrP deposits in the retina have not been characterized. Furthermore, a comparative analysis of PrP deposits occurring in the brain and in the retina in GSS has not been carried out. We hypothesize that the PrP aggregation and seeding properties in the brain and retina differ. The aim of this study was to analyze PrP from brains and retinas of individuals affected by GSS, all carriers of the *PRNP* F198S mutation from the same pedigree. Postmortem tissues from these patients were analyzed using neuropathologic and biochemical methods including Real-Time Quaking Induced Conversion assay. PrP deposits in the retina were found only in the outer plexiform layer, did not have tinctorial property of amyloid and were immunopositive using nine antibodies of epitopes throughout the PrP sequence. The biochemical analysis showed that PrP–F198S in the retina is partially detergent-insoluble, is assembled in large-size aggregates, and has seeding property. In addition, the glycosylation of full-length PrP in the retina was more complex than that of control PrP. Furthermore, the homogenates of retina did not contain the 8 kDa PrP internal fragments (PrP^IF^) as brain homogenates do; however, a PrP^IF^ was experimentally generated using proteinase K. In conclusion, we report for the first time the neuropathologic and biochemical properties of PrP in the retina of individuals carrying F198S and compare them with those in the brain of the same individuals. The fact that in GSS F198S, PrP^IF^ is constitutive only in the brain points to the need of identifying whether there is a difference in proteolytic mechanisms between the retina and brain.

## INTRODUCTION

Gerstmann-Sträussler-Scheinker disease (GSS) is an adult-onset dominantly inherited prion protein (PrP) amyloidosis caused by mutations in *PRNP* [12, 14–16, 27, 28, 54]. The *PRNP* missense mutation TTC to TCC at codon 198 results in the substitution of serine for phenylalanine at residue 198 (F198S) of PrP and causes a GSS variant (GSS F198S), that has been extensively studied in mutation carriers from a large kindred [7–9, 11, 13, 15, 16, 21, 22, 28, 30, 47, 48, 51, 63, 69, 70]. The polymorphism methionine/valine at residue 129 modifies the disease phenotype; more specifically, patients homozygous for valine at residue 129 have clinical signs more than 10 years earlier than heterozygous patients [9]. GSS F198S has a high penetrance and is clinically characterized by behavioral changes, cognitive impairment, cerebellar ataxia, parkinsonism and pyramidal signs. Neuro-ophthalmologic studies revealed the presence of abnormal eye movements that are related to the basal ganglia and cerebellar involvement [11, 69]. The clinical course may last from two to 15 years with symptoms starting between the fourth and eighth decade of life [16].

The neuropathologic phenotype of GSS F198S is characterized by the coexistence of aggregates of two misfolded proteins that, in histologic and immunohistochemical preparations, have a distinct morphology as observed by brightfield and fluorescence microscopy; the first is the extracellular unicentric and/or multicentric PrP deposit (plaque) and the second is the intraneuronal neurofibrillary tangle resulting from the aggregation of 3R/4R tau [13, 17, 21, 22]. PrP deposits and intracytoplasmic tau inclusions are both present in the cerebral cortex and subcortical nuclei; however, PrP deposits are present in the cerebellar cortex, but tau inclusions are not. PrP deposits are in the form of plaques with a single or multiple amyloid cores that are fluorescent using thioflavin S; the cores are surrounded by non-fluorescent diffuse material. By immunohistochemistry, PrP antibodies immunolabel both cores and the surrounding diffuse material.

Protein sequencing demonstrated that the core of the PrP plaques is made of PrP’s N- and C-terminally truncated fragments [48, 60, 61, 72]. Immunoblot analysis of proteinase K (PK)-digested homogenates of brain tissue has shown that PrP migrates as three prominent bands of ~28, ~18 and 8 kDa. The latter band corresponds to a monomeric PrP internal fragment (PrP^IF^) cleaved at the N- and C-terminals, while the other two bands correspond to multimers of the internal fragment. PrP^IF^ sequence spans from residues 74–78 to 142–152 and the 18 and 28 kDa bands represent covalently linked multimers of distinct sizes that result from the polymerization of the 8 kDa fragment [7, 60]. Studies of PrP in GSS F198S using cryogenic electron microscopy (cryo-EM) have shown the structure of filaments of PrP amyloid. The filaments are composed of dimeric, trimeric and tetrameric left-handed protofilaments with their protomers sharing a common protein spiral fold that consists of 62 amino acids and spans from glycine 80 to phenylalanine 141 [22].

It has been stated that several major neurodegenerative disorders have manifestations in the retina, suggesting that the eye is a ’window’ into the brain [35]. PrP^C^ is an essential component for visual processing and studies using PrP knockout mice have shown that the absence of PrP^C^ is associated with alterations of retinal functions [59]. Visual disturbances may be the initial manifestation of Creutzfeldt-Jakob disease (CJD) and some reports indicate that retinal alterations are seen in electroretinograms (ERGs) of affected patients [29, 50]. Whether in sporadic CJD, PrP^SC^ is associated with the changes observed using ERG requires further investigation. Additionally, information on the ERG changes associated with mutant PrP and on the neuropathology of the retina in genetically-determined PrP amyloidosis is lacking [20, 23, 25, 45, 52, 62, 65].

Single cell RNA data from the Human Protein Atlas, shows PRNP RNA expression in retinal bipolar cells which are present in both the OPL and IPL [1]. In sporadic, hereditary, iatrogenic and variant CJD, both inner and outer plexiform layers contain PrP aggregates regardless of the type of PrP^SC^ inclusions in the brain [24, 25, 62].

In the course of our studies, we postulated that the pathogenetic mechanisms of PrP aggregation in GSS F198S may differ between brain and retina. Thus our studies on the extent and distribution, biochemical properties, and seeding propensity of pathologic PrP in GSS F198S were carried out to determine: 1) whether PrP aggregates in the retina are amyloid and 2) whether the location of these aggregates in the retina is similar to that in another form of GSS (i.e. GSS A117V) and/or to that previously observed in CJD.

## MATERIALS AND METHODS

The current study of GSS F198S comparing the molecular neuropathologies of brain and retina represents the continuation of work carried out during the preceding four decades using brain tissue of patients from the same pedigree [13, 47, 60, 61]. All research was carried out in accordance with the Institutional Review Board guidelines at Indiana University School of Medicine, the Declaration of Helsinki [67] and the Additional Protocol on the Convention of Human Rights and Biomedicine Concerning Biomedical Research [10].

### Subjects: GSS and Controls

For this study, we have studied the retina and brain obtained postmortem from eight GSS F198S mutation carriers. A review of clinical records indicate that following a clinical diagnosis made by a neurologist, the course of the disease lasted between two and 13 years (Supplemental Anamnestic Data). The patients A, B, C, D, E, F, G, and H died at 58, 48, 44, 49, 53, 73, 63, and 60 years of age, respectively. Brains from these individuals, three males (subjects A, B, E) and five females (subjects C, D, F, G, H), and retinas from seven of these individuals (subjects A-G) were studied neuropathologically. Biochemistry of PrP was carried on subjects D and E. To extend knowledge on the neuropathology of retina in dominantly inherited PrP amyloidosis, we included results obtained from the retina of a single case (subject I) of GSS A117V. This female patient died at 45 years of age following a clinical course that lasted 5 years (Supplemental Anamnestic Data). For negative controls, brain and retina tissue were obtained from two patients: a 78-year-old male with Alzheimer disease (AD), and a 67-year-old male with Dementia with Lewy Body (DLB). As a positive control, the brain and retina were obtained from a 68-year-old male with sporadic CJD (sCJD) (MM/1).

### Molecular Genetic Analysis

DNA was isolated from fresh postmortem brain tissue of each of the nine GSS subjects using previously described methods [40]. The resulting DNA sequences were compared to the published *PRNP* sequence [34].

### Neuropathologic study of GSS F198S and GSS A117V

Autopsies of the nine GSS subjects were carried out five (A), four (B), eight (C), eleven (D), thirteen (E), eighteen (F), five (G), nine (H) and six (I) hours after death. The autopsies of the three control subjects were carried out five hours after death. Subjects A, C, D, E, F, H, and I had complete autopsies; the eyes were obtained from subjects A, C, D, E, F and I. For subjects B and G only the brain, spinal cord and eyes were obtained. The weight of the fresh brains were as follows: 1360 g (A), 1218 g (B), 1122 g (C), 1226 g (D), 1452 g (E), unknown (F), 1304 g (G), 970 g (H), and 960 g (I). The weight of the fresh brains of the control with DLB was 1420 g, of the AD and sCJD are unknown. Brains were hemisected using the method previously described [51]. Following fixation in a 10% formalin solution, the left cerebral and cerebellar hemispheres as well as the left half of the brainstem were sliced and tissue samples selected as previously reported [36].

For subjects A, B, C, both eyes were divided into four quadrants (temporal, superior, nasal, inferior) and fixed in formalin. For subjects D, E and I, one eye was fixed in formalin and the other was frozen and stored at −70° C. For subject F, only one eye, fixed in 4% paraformaldehyde in phosphate-buffered saline (PBS), was available for this study. From control subjects, one eye was fixed in formalin and the other was frozen. Samples from each subject were dehydrated in graded alcohols, cleared in xylene, and embedded in paraffin.

Brain and eye tissue were cut into eight-micron-thick sections; coronal hemispheric sections were cut into ten-micron-thick sections; sections of each quadrant of the eyes were cut into five-micron-thick sections. Histological staining was carried out using hematoxylin and eosin (H&E), and luxol fast blue (LFB); to detect amyloid plaques of PrP and amyloid-β (Aβ) as well as tau neurofibrillary tangles, thioflavin S was used. In addition, fluorescence imaging of protein aggregates was carried out using the following two luminescent conjugated oligothiophenes (LCOs) ligands: pentamer-formyl thiophene acetic acid (pFTAA), and the pentameric thiophene HS-84 [2, 5, 33, 41, 49, 57, 58]. For PrP immunohistochemical studies, the sections were pretreated with 88% formic acid for 15 minutes at room temperature [31]. Immunohistochemical studies were carried out using nine anti-PrP antibodies that recognize the N- and C-termini as well as the mid-region. Supplemental Figure 1 shows schematically the epitope region of PrP, which is recognized by each one of the nine antibodies. Commercially available antibodies 12F10 (monoclonal, PrP 142-160 Caymen, 1:800), 1E4 (monoclonal, PrP 97-106, Cell Sciences, 1:500), and 3F4 (monoclonal, PrP 109-112, Dr. R. Kascsak, 1:800 and Biolegend, 1:500) as well as the following antibodies generated in house: PrP 23-40, PrP 58-71, PrP 90-102, PrP 95-108, PrP 151-165, PrP 220-231 (polyclonal, Dr. P. Piccardo/ Dr. B. Ghetti, 1:100) were used for the immunohistochemistry of brain and retina [46]. Other antibodies were also used to label tau protein (monoclonal, AT8, Thermo Fisher; 1:200), Aβ protein (monoclonal, 21F12, Elan Pharmaceuticals; 1:1000, 4G8 monoclonal, BioLegend, 1:1000, NAB228 monoclonal, Invitrogen, 1:200), glial fibrillary acidic protein (polyclonal, Dako; 1:100), α-synuclein Asy119-137 (polyclonal, Dr. P. Piccardo/ Dr. B. Ghetti; 1:300), and Calbindin Dk28 (polyclonal, Swant, 1:3000), a calcium-binding antibody. Synaptophysin (polyclonal, Dako; 1:100) was also used to show, by immunohistochemistry, the outer (OPL) and inner plexiform layers (IPL) of the retina[42, 68].

Tissue sections were pretreated as needed for appropriate antigen retrieval and then were incubated overnight at room temperature for either single or double immunohistochemical labeling. For single labeling, using either a monoclonal or a polyclonal antibody, the ImmPRESS HRP anti-rabbit or anti-mouse IgG (Vector Labs, Burlingame, CA) antibody was used where appropriate, with the brown chromogen 3, 3’-diaminobenzidine for visualization. For labeling the retinas, Chromogen ImmPACT Vector Red alkaline phosphatase substrate (Vector Labs) was also used. For double labeling with the two antibodies Calbindin and 12F10, two chromogen were used in order to yield two colors: Calbindin labeling was prepared using the HRP/DAB modified-to-black chromogen; then, after rinsing, antibody 12F10 was applied and incubated overnight. Subsequently, labeling with 12F10 was obtained by the use of Vector ImmPRESS AP alkaline phosphatase anti-mouse IgG (Vector Labs), with the chromogen ImmPACT Vector Red alkaline phosphatase substrate (Vector Labs).

### Immunofluorescence to Determine the Colocalization of PrP with Retinal Markers

Superior calotte and nasal calotte retinas were fixed in formalin, embedded in paraffin, and then sectioned. Tissue sections were deparaffinized and antigen retrieval was performed in BioCare De-cloaker chamber in 0.01 M Citrate buffer at pH 6.0 for 20 minutes at 120°C/20 psi. Sections were blocked in 0.1 M Glycine in 0.01 M PBS for 30 min at room temperature followed by 1 hr in normal donkey serum blocking/dilution solution (2% donkey serum, 1% BSA, 0.1% Triton X-100, 0.05% Tween 20 in 0.01 M PBS). Primary antibodies were applied overnight at 4°C. Slides were incubated in Alexafluor 488 goat anti-rabbit and Alexafluor 568 goat anti-mouse secondary antibodies (ThermoFisher, 1:1000) and DAPI (5μg/mL) for 1 hr at room temperature and then coverslipped with ProLong Gold Antifade Mountant (Invitrogen). Commercially available antibodies were used to label C-terminal binding protein 2/ribeye protein of the ribbon synapse (polyclonal, Ctbp2, Invitrogen, 1:100), Protein kinase C (rabbit polyclonal, PKCα, Invitrogen 1:100) a rod bipolar cell antibody, Iba-1 (rabbit polyclonal, Biocare Medical, 1:200) a microglia antibody, and 3F4 (mouse monoclonal, 1:100).

### Confocal microscopy

Samples were imaged using a Nikon Ti2 microscope and NIS element software 6.02.03 (Nikon). A Plan Fluorite 40x/0.75 lens and High Power Apochromat Total Internal Reflection Fluorescence 100x/1.49 oil immersion lens were used, with an immersion oil at a refractive index of 1.518. Image analysis was done using ImageJ (NIH).

### Quantitative Analysis of PrP aggregates

Slides of frontal cortex, cerebellar cortex and retina from two cases (Cases D and E) were used for quantitative analysis. Using the Huron Tissuescope LE120 (Huron Digital Pathology, St. Jacobs, ON, Canada), digital images were created from slides stained with thioflavin S and immunolabeled with PrP antibody 3F4. Measurements were obtained using the Huron Viewer software. The maximum resolution of the Tissuescope LE120 is 0.3 um per pixel, which was used for this study. For each case, PrP burden was assessed by measuring an area of PrP immunopositivity within a defined tissue area of the frontal cortex and of the molecular layer of the cerebellar cortex and calculating the area.

Using immunohistochemical preparations with the 3F4 antibody, the diameter, circumference and surface area was measured for 60 plaques from the frontal cortex and the molecular layer of the cerebellar cortex as well as 60 beads from the retina. Additionally, using thioflavin S preparations, the diameter, circumference and surface area were measured for 60 plaques cores in the frontal cortex and molecular layer of the cerebellar cortex. Using the HuronViewer software, the diameters of plaques, cores and beads were obtained using the ruler tool. The Aperio Imagescope (Leica Biosystems, Deer Park, IL, USA) was used to obtain the surface area and circumference of each PrP aggregate.

### Biochemical analysis and sample preparation

Fresh tissues of frontal cortex and retina were dissolved in nine volumes of lysis buffer (100 mM NaCl, 10 mM EDTA, 0.5 % Nonidet P-40, 0.5 % sodium deoxycholate, 10 mM Tris pH 7.4) and clarified by centrifugation at 1,000 g for 10 min. The supernatant was stored at −80° C, whereas the pellet was discarded. Detergent-soluble and -insoluble fractions were obtained by two cycles of centrifugation at 100,000 g for 90 min, as previously described [38]. For N-deglycosylation, samples were treated with N-glycosidase F (PNGase F) according to the manifacturer’s instructions (Boehringer, Mannheim) for 8 hours at 37° C.

### Sedimentation velocity in sucrose gradient

Starting from the same amount of tissue, 100 μl of 20 % frontal cortex and retina homogenates were incubated with an equal volume of 2 % Sarkosyl for 30 min on ice. Samples were loaded atop a 10–60 % step sucrose gradient (300 μl each of 10 %, 15 %, 20 %, 25 % 30 % and 60 % sucrose) and centrifuged at 200,000 g for 1 h at 4° C, as described. Eleven fractions were collected from the top of the tube for Western blot analysis of PrP [38].

### Proteinase-K resistance assay

Protease resistance was assayed by incubating brain and retina samples with 50 and 20 μg/ml of Proteinase-K (Roche Applied Science), respectively, for 30 min at 37° C. To study the kinetics of protease resistance, brain and retina samples were treated with 20 μg/mL of PK, for 5, 10, 20, and 40 min and at each time point the proteolytic digestion was blocked by the addition of 3 mM phenylmethylsulfonylfluoride.

### SDS- and 2D-PAGE analyses

For SDS-PAGE, 10 % brain and retina tissue lysates were dissolved in sample buffer (3 % SDS, 3 % β-mercaptoethanol, 2 mM EDTA, 10 % glycerol, 62.5 mM Tris, pH 6.8) and boiled for 5 min. 10 ml of each sample were loaded onto 12 % SDS-PAGE gels. Proteins were transferred onto polyvinylidene difluoride membrane for 2 h at 60 V and membranes were blocked with 1 % nonfat dry milk in TBST (10 mM Tris, 150 mM NaCl, 0.1 % Tween 20, pH 7.5) for 1 h at 37° C. Membranes were incubated with anti-PrP monoclonal antibody 3F4 (1:10,000) or 6H4 (epitope 144–152) (Prionics) (1:5,000) overnight at 4° C. Blots were developed with an enhanced chemiluminescence system and PrP was visualized on autoradiography film. The two antibodies were selected because 3F4 recognizes the 8 kDa internal fragment and 6H4 does not [38]. The films were scanned with a high resolution Epson scanner and captured as 16-bit TIFF files. Densitometric quantification of the protein bands was performed using Un-Scan-It software (Silk Scientific, USA) and calculated as previously described [71].

For isoelectric focusing (IEF), pre-cast gels with a linear pH range of 3–10 were used (Biorad). Before IEF, the equivalent of 2 mg of wet tissue was resuspended in 125 μl of buffer (6 M urea, 2 M thiourea, 5 % β-mercaptoethanol, 2 % Nonidet P-40, and 2 % ampholyte) and sonicated for 2 min. Dry gels were reswollen in this solution for 15 h. IEF was carried out at 20° C for 4 h with raising voltage (500–8000 V), in a cooled horizontal electrophoresis unit (IPGphor, Pharmacia). For the second dimension, the IPG strips were equilibrated for 20 min in 50 mmol/L Tris-HCl, 6 M Urea, 10 % glycerol, 2 % SDS and a trace of bromophenol blue and loaded on SDS-PAGE. Immunoblotting was performed as described above and PrP was revealed with 3F4 (1:10.000).

### Recombinant PrP protein preparation

Recombinant prion protein (rPrPSen) substrates including recombinant full length hamster prion protein (rPrP^Sen^ 23–231), truncated hamster prion protein (rPrP^Sen^ 90–231) and bank vole prion protein (rPrP^Sen^ 90–231 V109M) were prepared, and purified as previously described [3, 43, 44].

### Real-Time Quaking-Induced Conversion (RT-QuIC) Assays

RT-QuIC reactions were carried out in subjects D and E as previously described [3]. Reaction mix was composed of 10 mM phosphate buffer (pH 7.4), 300 mM NaCl, 0.1 mg/ml hamster or bank vole rPrP^Sen^, 10 μM thioflavin T (ThT), 1 mM ethylenediaminetetraacetic acid tetrasodium salt (EDTA), and 0.001% SDS. Aliquots of the reaction mix (98 μL) were loaded into each well of a black 96-well plate with a clear bottom (Nunc) and seeded with 2 μL of retinal and brain homogenates at dilutions of 10^−2^, 10^−4^, and 10^−6^. The plate was then sealed with a plate sealer film (Nalgene Nunc International) and incubated at 55° C in a BMG FLUOstar Omega plate reader with cycles of 1 min shaking (700 rpm double orbital) and 1 min rest. ThT fluorescence measurements (450 +/−10 nm excitation and 480 +/−10 nm emission; bottom read) were taken every 45 min. Each sample was run in quadruplicate. A response of more than 3 standard deviations (3SD) above the mean of the negative controls in two or more of the replicates was considered positive. A Spearman-Kärber analysis was used to estimate a seeding dose or dilution at which 50% of the wells became ThT positive (SD_50_) as previously described [66].

## RESULTS

### Histopathologic and Immunohistochemical Studies of the Retina

Upon dissection, the eyes of subjects A, C, D, E, F, G and I were macroscopically unremarkable while those of subject B showed focal thickening and gray/white discoloration of the retina around the optic nerve head. Subject B had ocular histopathologic changes consistent with a clinical history of uncontrolled diabetes mellitus, including thickening of arteriolar walls and basement membranes. In subjects A, C, D, E, and F, retinopathy was not observed. None of the subjects had inflammatory infiltrates or loss of any cellular layers in the retina.

**Figure 1 -:**
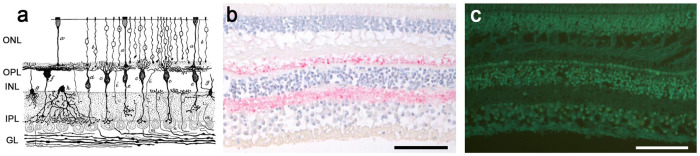
Human retina stained with three different techniques. Drawing by Santiago Ramón y Cajal in 1892 of a human retina from a Golgi preparation illustrating, vertically from the bottom, the retinal layers: optic fiber, ganglion cell, inner plexiform, inner nuclear, outer plexiform, outer nuclear (a); the inner and outer plexiform layers immunolabeled using synaptophysin (b); a thioflavin S preparation shows non-fluorescent bead-like deposits in the retina of an individual affected by GSS F198S. Bars: 100 μm (b-c). Subject: D (b-c).

Ramon J. Cajal’s original drawing shows the organization of the architecture of the normal human retina as seen in a Golgi preparation and it is used in parallel with pictures of the immunohistochemical preparation of retina labeled by antibodies to synaptophysin and of a thioflavin S preparation (Fig. 1 a–c). In GSS F198S subjects, the OPL and IPL were decorated by the synaptophysin antibodies; non-fluorescent bead-like structures are seen in the OPL in the thioflavin S preparation (Fig. 1 b–c).

**Figure 2 -:**
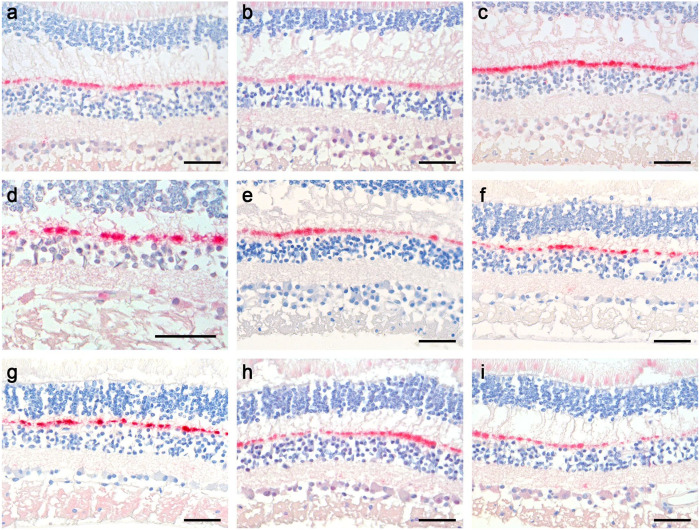
Immunohistochemistry of PrP inclusions in the retina of an individuals with GSS F198S. PrP immunopositive bead-like deposits are located in the outer plexiform layer (OPL) and labeled with anti-PrP antibodies: PrP 23-41 (a), PrP 58-71 (b), PrP 90-102 (c), PrP 95-108 (d), 1E4 (PrP 97-106) (e), 3F4 (PrP 109-112) (f), 12F10 (PrP 142-160) (g), PrP 151-165 (h), PrP 220-231 (i). Bars: 50 μm (a-i). Subject: D (a-i).

**Figure 3 –:**
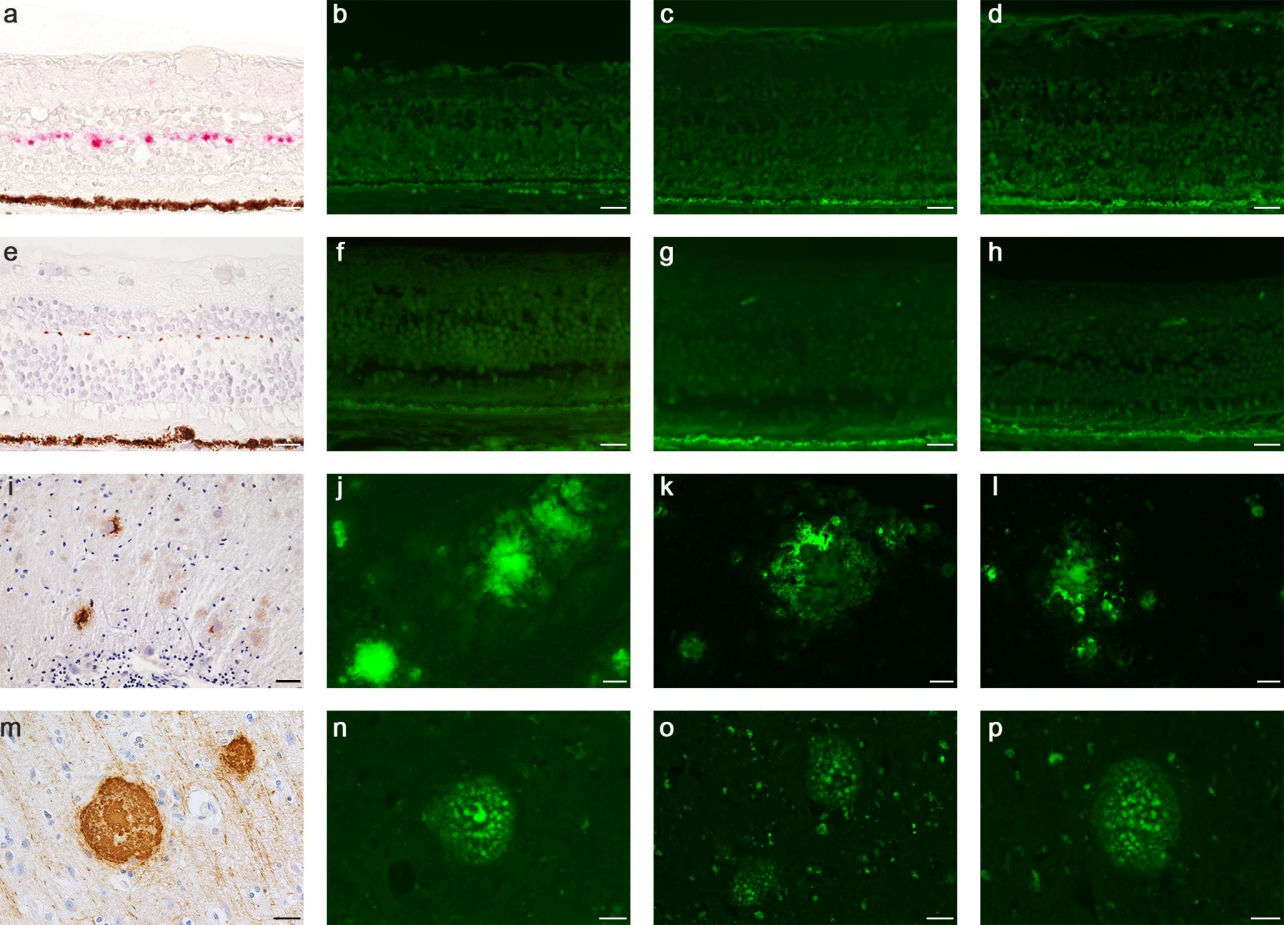
Retina and Brain from individuals with GSS F198S and GSS A117V. PrP-immunopositive bead-like deposits are located in the OPL and labeled anti-PrP antibody 3F4 (PrP 109-112) in a patient with GSS F198S (a). In thioflavin S preparation, PrP deposits are not fluorescent in a patient with GSS F198S (b). In sections labeled with pFTAA, PrP deposits are not fluorescent in a patient with GSS F198S (c). In sections labeled with HS-84, PrP deposits are not fluorescent in a patient with GSS F198S (d). PrP-immunopositive bead-like deposits are located in the OPL and labeled anti-PrP antibody 12F10 (PrP 142-160) in a patient with GSS A117V (e). In thioflavin S preparation, PrP deposits are not fluorescent in a patient with GSS A117V (f). In sections labeled with pFTAA, PrP deposits are not fluorescent in a patient with GSS A117V (g). In sections labeled with HS-84, PrP deposits are not fluorescent in a patient with GSS A117V (h). Aβ-immunopositive deposits labeled with NAB228 are seen surrounding cores of PrP plaques in the cerebellar cortex of a patient with GSS F198S (i). Thioflavin S preparation shows a large fluorescent aggregate, but does not distinguish the nature of the fluorescent aggregate in the cerebellar cortex of a patient with GSS F198S (j). pFTAA and HS-84 preparation reveal fluorescence surrounding the PrP plaques in the cerebellar cortex of a patient with GSS F198S (k-l). PrP-immunopositive plaque labeled by anti-PrP antibody 3F4 (109–112) in the frontal cortex of a patient with GSS A117V (m). Thioflavin S preparation shows a large fluorescent multicentric plaque in the frontal cortex of a patient with GSS A117V (n). pFTAA and HS-84 show fluorescent multicentric plaques in the frontal cortex of a patient with GSS A117V (o-p). Bars: 25 μm (a-h, j-p); 50 μm (i). Subject: D (a-d, i-l), I (e-h, m-p).

The immunohistochemical studies using antibody 3F4 showed that the bead-like deposits seen in thioflavin S preparations in OPL were immunopositive for PrP in the retina of seven GSS F198S subjects (subjects A-G). The beads in subjects D and E, immunolabeled with 3F4, had an average diameter of 7.75 μm (±1.93 μm, range = 3.80 – 17.50 μm), an average circumference of 30.55 μm (±6.49 μm, range = 18.42 – 62.48 μm), and an average surface of 52.04 μm^2^ (±23.40 μm^2^, range = 19.22 – 180.68 μm). In subject D, the bead-like deposits in the OPL were decorated by PrP antibodies directed to nine different epitopes (23–40, 58–71, 90–102, 95–108, 97–106, 109–112, 142–160, 151–165, 220–231) spanning the entire PrP sequence (Fig. 2 a–i). PrP immunopositivity was not seen in the cornea, sclera, lens, iris, ciliary body, choroid, or optic nerve. Immunohistochemical studies using 12F10 in the GSS A117V case revealed the presence of immunopositive bead-like deposits in the OPL. PrP immunoreactivity was negative in the AD and DLB control retinas. In the retina of GSS A117V, fluorescence was not detected in thioflavin S and LCO preparations (Fig. 3 a–h). Antibodies to tau, amyloid β (Aβ), α-synuclein, and TDP-43 did not decorate any layer of the retina (data not shown).

**Figure 4 -:**
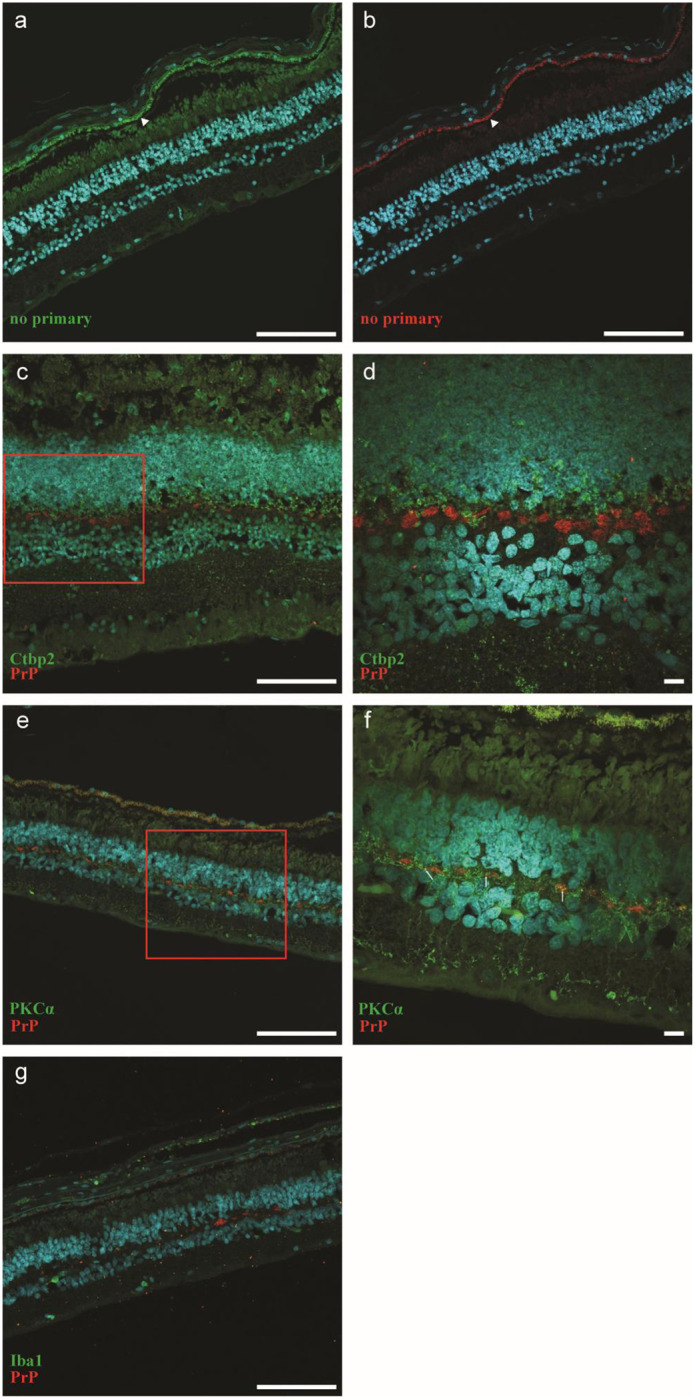
PrP co-localizes with ribbon synapse marker and with rod bipolar cell marker. Nasal calotte retina (a-f) stained with secondary antibody only (no primary) (a-b), and 3F4 co-stained with ribbon synapse marker Ctbp2 (c-d) or rod bipolar cell marker PKCα (e-f). Non-specific staining of PrP, Ctbp2, and PKCα observed in retinal pigment epithelium (RPE), indicated by white arrowhead (a-b). Bead-like PrP deposits (red) localized in outer plexiform layer (OPL) close to the ribbon synapses (green) (c-d). Magnification of boxed area in panel c (d). Co-staining of PrP and rod-bipolar cells (e-f). Magnification of boxed area in panel e (f). White arrows indicate PrP colocalization with rod bipolar dendrites (f). Co-staining of PrP and microglia (g). In all panels nuclei were stained with DAPI. Bars: 100 μm (a-c, e, g), 10 μm (d,f). Subjects: D (a-g).

To further elucidate the localization of the bead-like PrP deposits, retinal sections from two symptomatic individuals (Subjects D, E) were immunostained with PrP and retinal cell markers (Fig. 4 a–g and Supplemental Fig. 2 a-e). PrP and retinal cell marker non-specific staining was observed in the retinal pigmented epithelium (RPE) (Fig. 4 a–b). Co-immunostaining of PrP and Ctbp2, a ribbon synapse marker, indicated PrP localized to the OPL and concentrated in the synapse in the area where the dendrites of horizontal and bipolar cells invaginate photoreceptor cells (Fig. 4 c–d and Supplemental Fig 3 a-b). Co-immunostaining of PrP and PKCα, a rod bipolar cell marker, also showed that the PrP colocalized with the dendrites of the rod bipolar cells (Fig. 4 e–f and Supplemental Fig. 2 c-d). Co-immunostaining of PrP and the microglia marker Iba1 was inconsistent with gliosis and microglial cell activation, with no co-localization observed between PrP and microglia (Fig. 4 g and Supplemental Fig. 2 e).

### Histopathologic and Immunohistochemical Studies of the Brain

In GSS F198S mutation carriers, coronal histological preparations of the cerebral hemispheres, stained with LFB-H&E, reveal a pallor of the white matter of the centrum semiovale due to the loss of myelinated fibers (Supplemental Fig. 3 a). The neuropathology in the cerebral cortex and subcortical nuclei is dominated by the presence of PrP and tau immunopositivity (Supplemental Fig. 3 b-c); however, it is noteworthy that the lateral geniculate nucleus (LGN) was free of tau and PrP deposits (Supplemental Fig. 4 a-c). A sagittal section of the cerebellar vermis reveals the presence of numerous thioflavin S fluorescent deposits (Supplemental Fig. 5 a). Parasagittal sections reveal a diffuse cortical atrophy and severe PrP immunopositivity of the cerebellar cortex (Supplemental Fig. 5 b-c).

**Figure 5 -:**
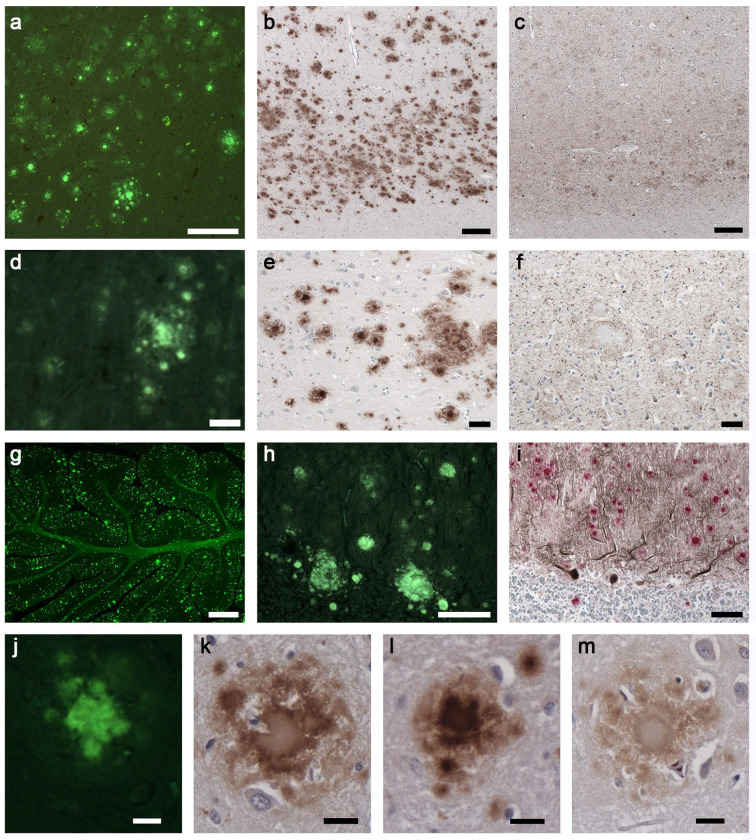
PrP and tau pathology in the cerebral and cerebellar cortices from affected individuals with GSS F198S. Neocortex (a-f, j-m) stained with thioflavin S (a,d,j) and immunolabelled with 3F4 (b,e), AT8 (c,f), PrP 23-40 (k), PrP 90-108 (l), and PrP 220-231 (m). Cerebellar cortex (g-i) stained with thioflavin S (g-h) and double immunolabeled with 3F4 (red) and calbindin (brown) (i). In the neocortex, fluorescent plaques are seen (a) and many of them have multiple cores; the amyloid plaques are PrP-immunopositive (b) and at higher magnification (e) the core and the diffuse material can be seen; tau-immunopositivity of nerve cell bodies and neurites is most prominent in deep cortical layers (c,f). In the cerebellum, fluorescent plaques are seen at different magnifications (g-h) and immunopositive PrP plaque cores are visible within the calbindin immunopositive dendrites of the Purkinje cells (i). In the neocortex, different views of plaques are seen (j-m): a fluorescent plaque core (j), a plaque labeled by an antibody to the PrP amino terminus (k), labeled by an antibody to the PrP mid-region (l) and labeled by antibody to the PrP carboxy terminus (m). Bars: 200 μm (a-c), 50 μm (d-f, i), 1 mm (g), 100 μm (h), 20 μm (j-m). Subjects: A (b-c,e-f,i), C (a,d,h), E (g, j-m).

In the cerebral cortex and subcortical nuclei, atrophy, nerve cell loss, and gliosis are present with different degrees of severity. PrP immunopositive deposits include unicentric and multicentric plaques as well as diffuse deposits. The unicentric and multicentric plaques are composed by a core and surrounding diffuse deposits. The cores and the diffuse deposits have different tinctorial properties; in fact, the cores are fluorescent in thioflavin S preparations while the diffuse deposits are not fluorescent (Fig. 5 a–b, d–e, g–h, j–m). PrP and tau pathologies are most prominent in the deep layers of the cerebral cortices (Fig. 5 a–f). PrP amyloid cores, when surrounded by tau immunopositive neurites (Fig. 5 f), are reminiscent of the neuritic plaque seen in AD. While the core of the plaques was immunolabeled by antibodies with epitopes between residues 80–150, that correspond to the sequence of the 8 kDa fragment, the periphery of PrP plaques were decorated only by PrP antibodies directed to the N- and C-termini (Fig. 5 j–m). The cerebellum was severely affected by the presence of numerous amyloid plaques made of PrP immunopositive deposits (Fig. 5 g–I and Supplemental Fig. 5 a, c). A consistent finding was the absence of any tau immunopositivity in the cerebellar cortex and in the cerebellar nuclei. Aβ-immunopositive plaques are not present; however, Aβ immunopositivity was seen surrounding PrP cores as previously reported [4]. Double immunohistochemistry for Calbindin and PrP showed that within the cerebellar cortex the loss of Purkinje cells and their dendrites corresponded to areas of the molecular layer in which amyloid plaques were most numerous (Fig. 5 i). In the cerebellum of GSS F198S, LCOs HS-84 and pFTAA revealed fluorescence of aggregates surrounding plaque cores. This finding is consistent to that observed using immunohistochemistry for Aβ (Fig. 3 i, m). The cores of the plaques were fluorescent with thioflavin S and visible, but not fluorescent, with the two LCOs (Fig. 3 j–l, n–p). Sections from the spinal cord reveal occasional PrP deposits in the dorsal horns and a moderate loss of myelinated axons in the lateral corticospinal tracts (data not shown).

The burden represented by immunopositivity of PrP in the molecular layer of the cerebellar cortex and frontal cortex was found to average 35.5 % and 16.9 % of the tissue area, respectively. In the frontal cortex, plaques had an average diameter of 148.46 μm (±49.82 μm, range = 75.8 – 412.4 μm), an average circumference of 421.96 μm (±122.30 μm, range = 125.3 – 896.75 μm), and an average area of 10,423.56 μm^2^ (±585.55 μm^2^, 3,649.23 – 385,358.41 μm). In the molecular layer of the cerebellar cortex, the plaques had an average diameter of 175.02 μm (± 56.11μm, range = 87.6 – 399.4 μm), an average circumference of 523.37 μm (±182.39 μm, range = 197.27 – 1,210.1 μm), and an average surface area of 17,258.99 μm^2^ (±14,206.90 μm^2^ range = 2,414.3 – 92,905.25 μm).

We also compared the core of the amyloid plaques in the frontal cortex with those of the molecular layer of the cerebellar cortex. In the frontal cortex, cores had an average diameter of 22.51 μm (±7.13 μm, range - 11.3 – 43.0 μm), an average circumference of 87.48 μm (±24.72 μm, range = 47.56 – 170.69 μm), and an average area of 477.48 μm^2^ (±279.64 μm^2^, range = 125.91 – 1,758.7 μm). In the molecular layer of the cerebellar cortex, the cores had an average diameter of 32.13 μm (±9.35 μm, range = 14.4 – 58.4 μm), an average circumference of 112.23 μm (±30.34 μm, range = 63.76 – 201.78 μm), and an average surface area of 785.09 μm^2^ (±423.13 μm^2^, range = 237.78 – 2,429.8 μm).

### Biochemical characterization of detergent-soluble and insoluble PrP in the brain and the retina

**Figure 6 -:**
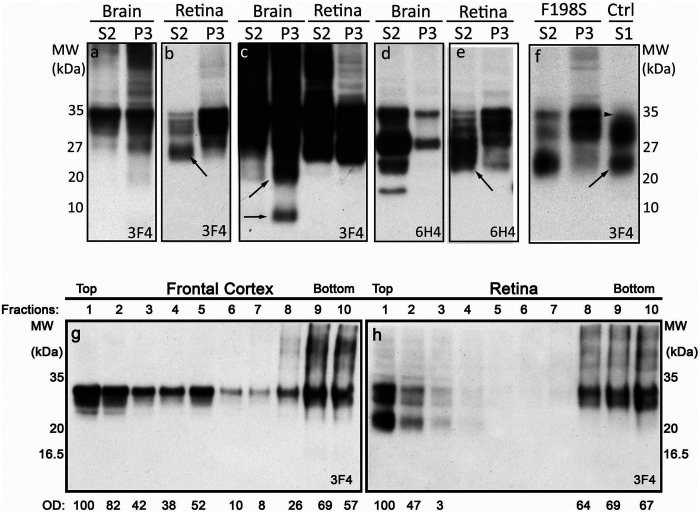
Immunoblot analyses with 3F4 and 6H4 of detergent-soluble (S2) and detergent-insoluble fractions (P3) and sucrose gradient separation of retinal and frontal cortex preparations. In brain homogenate three major PrP bands of 35, 30 and 27 kDa representing the di- mono- and unglycosylated PrP (a). Two faster migrating bands at 8 and 20 kDa were found in P3 in overexposed blots (c, arrows). Retina sample preparations showed PrP indistinct bands between 35 and 30 kDa and a broad band of 25 kDa (S2, arrow) while in P3 the 35 and 30 kDa bands were highly represented (b). Immunoblot with 6H4 of brain homogenate, showed in S2 four major bands of 35, 27, 20 and 18 kDa (d) and by two bands of 35 and 27 kDa in P3. In S2, retina homogenate separated as several bands between 35 and 25 kDa while in P3 indistinct bands were seen between 27 and 35 kDa (e). Immunoblot analysis of S2 and P3 from F198S and total retina homogenate from control (f). In control retina, PrP separated as two broad bands of 30 and 25 kDa (arrow); unlike F198S preparation, the highly glycosylated PrP bands of around 35 kDa are almost absent (f, arrowhead); (g, h) in sucrose gradient preparation of frontal cortex, PrP was mainly found on top (1–5) and bottom fractions. In fractions 8–10, large size aggregates of PrP migrate in the range of 25 to 35 kDa (g); in the retina PrP is detected in the first two (1–2) and bottom fractions (8–10). Following film over exposition, the 8 kDa fragment was seen only in the brain at the bottom fraction but not in the retina (data not shown). At the bottom of the immunoblots is reported the relative percentage of total PrP in each lane (considering fraction 1 as 100% with highest amount of PrP in both brain and retina). To provide a direct comparison between brain and the retina, total PrP optical density at lane 1 is identical in both brain and retina.

To characterize the properties of PrP in brain and retina, we first analyzed the glycosylation pattern of PrP in the detergent soluble (S2) and insoluble (P3) fractions. PrP immunoblot analysis of the detergent-soluble S2 fraction with 3F4 showed three major PrP bands migrating at 35, 30, and 27 kDa, representing the di-glycosylated, mono-glycosylated, and unglycosylated full-length PrP isoforms, respectively. In the retina, the PrP banding pattern was characterized by indistinct bands between 35 and 30 kDa and by a broad band migrating at 25 kDa which was not observed in brain (Fig. 6 a–b). By contrast, in the detergent-insoluble P3 fractions, the PrP glycosylation pattern was similar in both brain and retina except for two bands of 20 and 8 kDa that were only observed in the brain following over-exposure of the blots (Fig. 6 c arrows, d). Following PNGase-F treatment, PrP was reduced to a single major band of 27 kDa in the S2 and P3 fractions in both brain and retina (Supplemental Fig. 6 a-d).

Immunoblot analysis of the same brain and retina samples with the anti-PrP 6H4 antibody was carried out in order to better define the contribution of PrP peptides containing the C-terminus. In brain, the S2 fraction showed a band at 35 kDa (i.e. full-length glycosylated PrP), and three other bands at 27, 22 and 18 kDa that were not detected by the 3F4 antibody. These bands represent the glycosylated isoforms of PrP C-terminal fragments and were not observed in the P3 fraction (Fig. 6 d). Following PNGase-F treatment, three PrP bands of 27, 20, and 18 kDa corresponding to full length PrP and the “C1” and “C2” C-terminal fragments [6], were detected. These bands were more prominent in the S2 fraction when compared to the P3 fraction (Supplemental Fig. 6 a-d). In the retina, the PrP banding pattern detected by 6H4 in both the S2 and P3 fractions was similar to that observed by 3F4 (Fig. 6 e). Following deglycosylation, PrP in the retina resolved in the P3 fraction as a faint band of 27 kDa (full length PrP) and an 18 kDa band (C1 fragment), while in S2 a 20 kDa band was also observed (Supplemental Fig. 6 a-d).

Unglycosylated full-length PrP migrates as a 27 kDa band in both brain and retina, with the N-terminally truncated fragments C1 and C2 also having the same migration pattern in both tissues. However, PrP glycosylation in the retina was distinct from that of the brain in blots labeled with 6H4 (Fig. 6 d–e), with an additional band of 25 kDa detected specifically in the retina (Fig. 6 b, e arrow and f). To evaluate the potential influence of the F198S PrP mutation on retinal PrP glycosylation, we compared retinal S2 and P3 fractions from F198S homogenates with retinal homogenates from control tissue. In the control sample, PrP was characterized by two major smears of ~32–27 kDa and 25 kDa with higher bands migrating at 35 kDa not detectable (Fig. 6 f arrowhead). Thus, our data show that PrP in F198S retina is composed of full-length, detergent-insoluble glycosylated PrP with a glycosylation pattern distinct from that of control retina.

The distribution of PrP^IF^, the 8kDa fragment which spans PrP residues ~78–152, and its multimers which migrate at higher molecular weights, was determined in S2 and P3 brain and retina fractions by comparing the relative percentage of total PrP detected by 3F4 versus PrP detected by the 6H4 antibody, which does not recognize PrP^IF^. In brain immunoblotted with 3F4, 40% of PrP was in the S2 fraction and 60% in the P3 fraction, while in the retina 35% was in S2 and 65% in P3 (Fig. 6 a–b). In brain fractions immunoblotted with 6H4, the relative amount of PrP in S2 and P3 was drastically different, with 98% in the S2 fraction and only 2% in the P3 fraction. By contrast, in retina immunoblotted with 6H4, PrP was distributed similarly to that detected by 3F4, with 49% in the S2 and 51% in the P3 fractions. Indeed, these results indicate that detergent-insoluble PrP is composed of pathological PrP fragments in brain versus retina, with detergent insoluble PrP^IF^ produced in the brain, but not the retina.

### Sucrose-gradient fractionation of PrP^Sc^ aggregates in frontal cortex and retina

To determine the size of PrP aggregates in the brain and retina, homogenates were analyzed by sucrose gradient sedimentation. Immunoblotting using 3F4 showed that brain PrP sedimented in all fractions, but was more concentrated in the top (fractions 1–5), containing the smallest PrP aggregates, and bottom (8–10) fractions, containing the largest PrP aggregates (Fig. 6 g). The 8 kDa PrP^IF^ band was detectable in the bottom fractions only after overexposure of the blot (data not shown). In retina, distribution of PrP was more limited, with PrP detectable only in the top (1–2) and bottom (8–10) fractions. In the retina, PrP separated in the top fractions as a smear of 30–35 kDa as well as a broad 25 kDa band, while in the bottom fractions PrP was represented by two bands of 35 and 30 kDa and no 25 kDa band was detectable (Fig. 6 h). The distribution of PrP aggregates in brain and retina was determined by the relative percentage of total PrP in the individual fractions by densitometric analysis, with fraction 1 being considered the lane with the highest value. This qualitative and quantitative difference of PrP aggregates in brain and retina is demonstrated by the relative amount of total PrP in each lane (Fig. 6 g–h). These findings show that in brain preparations, PrP aggregates are of different sizes and detected throughout the gradient, while in retina preparations, they are exclusively detected in the top and bottom fractions (Fig. 6 g–h). Thus, the sucrose gradients provide a qualitative characterization of detergent-soluble and detergent-insoluble PrP in brain and retina. In particular, in retinal preparations the detergent soluble 25 kDa band detected in S2 was only found in the top fractions of the gradient, while the detergent-insoluble 30 and 35 kDa bands were found only in the bottom fractions.

### RT-QuIC assay on brain tissues and retina samples

**Figure 7 -:**
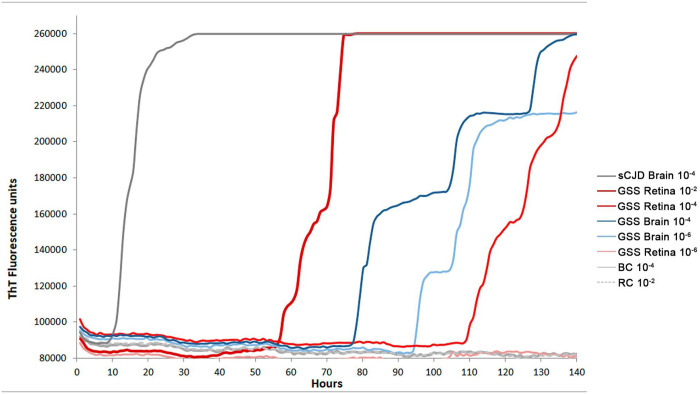
RT-QuIC analysis of brain and retina from patients with GSS F198S, using recPrP^sen^ from bank vole 90–231 V109M as the substrate of replication. In GSS F198S, PrP seeding activity is observed in brain tissue up to 10^−6^ dilutions. At a dilution of 10^−4^(dark blue line), the lag phase is 75 hours whereas at a dilution of 10^−6^ (light blue line) is 90 hours. Positive control brain tissue from a sporadic CJD case (sCJD), diluted to 10^−4^ (dark gray line), showed a lag phase of less than 10 hours and reached the plateau after 10 hours. In GSS F198S, retina samples were positive up to 10^−4^ dilutions; the lag phase was at 55 h at 10^−2^ (dark red line) and 110 h at 10^−4^ (red line) dilutions with the end-point dilution occurring at 10^−6^ (orange line). No seeding reactions were observed after 140 h in 10^−4^ diluted brain tissue from an Alzheimer disease (BC) (light gray line) and in 10^−2^ diluted retina control tissue (RC) from a case of dementia with Lewy body (dotted light gray line). Subjects D and E.

To further evaluate the properties of abnormal PrP in GSS F198S, we assayed PrP seeding activity using the RT-QuIC assay in fraction 1 obtained from sucrose gradient in brain and retinal samples from two subjects (D and E). Brain tissue from sCJD and AD cases were used as positive and negative controls, respectively. The retina from an individual whose diagnosis had been neuropathologically confirmed as DLB was also used as a negative control. RT-QuIC assays using either hamster rPrP^Sen^ 23-231 PrP at 42° C or hamster rPrP^Sen^ 90-231 at 55° C as reaction substrates did not show PrP seeding activity in either brain or retinal preparations (data not shown). Conversely, using recombinant PrP from bank voles (rPrP^Sen^ 90-231 V109M) at 55° C as a reaction substrate, a 10^−4^ dilution of brain homogenate was positive at 75 h, with PrP seeding activity detectable down to a 10^−6^ dilution with a lag phase of 95 hours (Fig. 7). In the retinal samples, PrP seeding reactivity was observed by 55 h in 10^−2^ diluted samples and at 110 h for 10^−4^ dilutions. No seeding activity was observed in retina at the end point dilution of 10^−6^. Seeding activity was not observed in control brain and retina up to 140 h (Fig. 7). An end-point dilution RT-QuIC analysis of brain and retinal tissue samples indicated that the concentration of seeding doses resulting in 50% ThT-positive replicate reactions (SD_50_) for these samples were 7.25 log_10_ SD_50_ per milligram of brain tissue and 5.25 log_10_ SD_50_ per microliter of retina samples. Thus, our results indicate that F198S retinal samples contain less PrP seeding activity than brain.

### Proteinase K-resistant PrP in detergent-soluble and insoluble fractions

**Figure 8 -:**
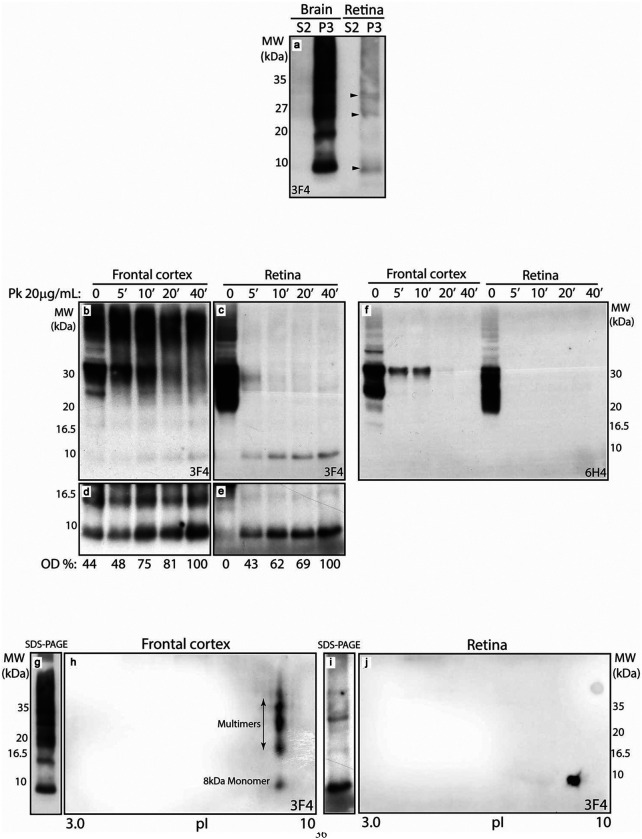
Immunoblot analysis of constitutive and PK generated 8 kDa internal fragment in brain and retina. Proteinase K-resistant PrP is shown in detergent-insoluble (P3) fractions but not in detergent-soluble (S2) fractions. In P3 fractions, the amount of PrP^res^ in brain tissue was consistently higher as compared to retina and PrP was resolved as a smear, except for three bands of 25, 16 and 8 kDa. In the retina, three faint bands of 30, 25 and 8 kDa were observed (a, arrow heads). Kinetics of PK-resistance of brain and retina preparations are shown in b-f. Brain and retina samples were digested with 20 μg/ml of PK for 5, 10, 20, and 40 min at 37° C (b and c represent gel at low exposure while panels d and e after long exposure). In frontal cortex, at time 0, low molecular weight bands were observed; following protease digestion higher migrating bands progressively decreased but are still detectable at 40 min (b) while the 8 kDa band increases progressively (d). The OD% reported below correspond to the relative percentage of 8 kDa band, PrP considering 100% the highest value at 40 min. In retinal samples, at time 0, PrP is composed by several bands migrating at 25–35 kDa and no lower bands were seen after gel overexposition (c, e); after 10 min of PK digestion, higher bands are consistently decreased while the 8 kDa band incremented (c, e). Immunoblot with 6H4, shows major PrP bands of 25–35 kDa in both retina and brain but not lower bands (f). Immunoblot with 3F4 of PK-treated frontal cortex and retina preparation following SDS- (g, i) and 2D-PAGE analysis (h, j). In frontal cortex, SDS-PAGE shows indistinct bands (g) while 2D-PrP pattern is characterized by series of spots with the same pI of ~9.0 and molecular mass of 8 kDa, corresponding to monomer of the internal fragment, and of 20, 30 and 35 kDa multimers (h); in SDS-PAGE separation retina samples shows a major band of 8 kDa (i); following 2D-PAGE, PrP resumes to a single spot with pI of ~9.0 and of 8 kDa (j).

We assessed the presence of protease resistant-PrP (PrP^res^) in S2 and P3 fractions in both brain and retina homogenates by exposing samples to 20 μg/mL of PK for 30 minutes at 37° C. Although in both brain and retina S2 PrP^res^ was undetectable, in P3 fractions, the amount of PrP^res^ in brain tissue was consistently higher when compared to the retina. In brain homogenates, PrP resolved as a smear, except for three bands of 25, 16 and 8 kDa which were recognized. In the retina, three faint bands of 30, 25 and 8 kDa were observed, with the last two migrating similarly to those found in brain tissue (Fig. 8 a).

### Kinetics of protease-resistance of PrP^c^ in brain and retina

To provide additional insights into the properties of retinal PrP, we analyzed the kinetics of PrP resistance to PK treatment. Frontal cortex brain homogenate and retinal homogenate were PK-digested (20 μg/mL) for 5, 10, 20 and 40 min at 37° C and total PrP detected using the 3F4 antibody. In the frontal cortex at time 0, PrP was represented by full length PrP isoforms ranging from 25 to 35 kDa and by lower bands at 8 and 16 kDa clearly visible in over-exposed blots (Fig. 8 b–c). By prolonging PK digestion, a progressive decrease of PrP bands migrating at 35–25 kDa was observed in conjunctions with a gradual increase of the 8 kDa, but not the 16kDa, band (Fig. 8 c).

At time 0, total PrP in the retina detected by 3F4 was characterized by high molecular weight bands between 35 and 27 kDa, as well as by a 25 kDa band. An 8 kDa band was not detectable (Fig. 8 e). The kinetics of PK digestion were similar to that observed in brain tissue, with an 8 kDa band appearing after 5 minutes of PK treatment and progressively increasing over time accompanied by a progressive decrease of the bands migrating at 35–25 kDa. The 16 kDa band was barely detectable (Fig. 8 e). Immunoblot analysis with 6H4 of both brain and retina homogenates showed full length PrP isoforms of 35–25 kDa which gradually decreased following PK digestion (Fig. 8 f). Overall, the data are consistent with the 8 kDa protease resistant PrP being generated in the brain, but not the retina, of F198S GSS.

### 2D-PAGE immunoblot characterization of F198S PK resistant-PrP in frontal cortex and retina preparations

To definitively demonstrate the composition of PK-resistant PrP in brain and retina, we used 2D-PAGE analysis to separate PrP based on its molecular mass and isoelectric point. Frontal cortex preparations showed that PrP^res^ was represented by multiple spots with the same isoelectric point (pI) of ~9.0 but with molecular masses of 8 kDa and ~16 to 35 kDa (Fig. 8 h). Based on pI and molecular mass, the 8 kDa spot corresponded to the monomer of PrP^IF^ (aa. ~80–150), while the higher molecular weight spots with the same pIs corresponded to PrP^IF^ multimers which were not entirely dissolved into 8kDa monomer, even in presence of high molar urea (Fig. 8 h). In the 2D-PAGE retinal preparations, PrP^res^ separated as a single and intense spot with a pI of 9.0 and a molecular mass of 8kDa corresponding to the monomer of the PrP^IF^ fragment (Fig. 8 j). When compared to the SDS-PAGE preparation of retina where faint bands of 30, 25 and 16 kDa were visible (Fig. 8 i), it’s likely that treatment with high molar urea in 2D-PAGE led to these retinal PrP^res^ bands being dissociated into a single 8 kDa monomer. These findings indicate that highly denaturing conditions dissolve PrP fragments generated *de novo* by PK digestion of PrP in the retina, but not the brain.

## DISCUSSION

The current study has addressed the issue as to whether in the retina, a developmentally external component of the CNS, the genetically mutated PrP presents the same pathology as that occuring in the brain. In many neurodegenerative diseases, the neuropathologic phenotypes at the level of the retina is not well known, even though the retina is part of the CNS [35]. However, it is important to emphasize that in spite of its layered structure, the architectural organization, synaptic connectivity, physiological and biochemical properties of the retina differ substantially from those of the brain. We hypothesized that different pathogenetic mechanisms lead to different modalities of PrP aggregation in brain versus retina. We tested this hypothesis by carrying out studies directed toward determining the extent and distribution, the biochemical properties, and the seeding propensity of misfolded PrP from affected individuals that belong to a single pedigree.

We studied *PRNP* F198S symptomatic carriers and, for the first time, we describe the PrP pathologic changes affecting the retina and compare them with the pathologic changes occurring in the brain. The present work represents a foundation toward an understanding of the pathogenesis of a dominantly inherited neurodegenerative disease caused by a mutation in the *PRNP* gene and has provided novel insights into regional differences of PrP aggregation in the CNS. The present study provides results that support the following interpretations related to the morphological and biochemical features of PrP deposits in brain and retina: (a) in the retina of GSS F198S, PrP deposits do not have the tinctorial properties of amyloid unlike the cores of the plaques in the brain; (b) in the retina of GSS F198S and A117V, PrP aggregates are detected only in the OPL; (c) the utilization of two LCOs has confirmed that PrP deposits in the retina do not have the tinctorial properties of amyloid; (d) in LCO preparations of GSS F198S cerebellum, the cores of the plaques can be visualized, but they are not fluorescent, while in LCO preparations of GSS A117V neocortex, the cores of the plaques are fluorescent; (e) deposits are located within the region in which synaptic connections occur between photoreceptor cells and other retinal cells; (f) a PrP 8 kDa internal fragment is constitutive in the brain, but not in the retina; (g) *ex vivo* PK digestion generates *de novo* the 8 kDa internal fragment in retinal tissue; (h) PrP from retinal tissue is composed by full length PrP, by C1 and C2, C-terminal fragments and by a C-terminal glycosylated fragment of 25 kDa that is not detected in the brain; (i) PrP in retinal tissue is composed of PrP aggregates with different sizes as compared to those in brain tissue; and (j) PrP from retinal tissue has lower seeding activity than PrP from brain tissue. These results provide the basis for inquiring about the posttranslational modifications of misfolded PrP in GSS F198S and A117V in different parts of the CNS. Future studies, designed to understand the pathogenetic mechanisms leading to the formation of PrP amyloid in the brain and those that prevent the formation of amyloid in the retina, will also need to take into consideration the distinct differences existing between the blood-brain barrier and the blood-retinal barrier, the regional differences in gene expression, as well as the differences in the local cellular and molecular environments. The data obtained have a broad biological significance since the differences between retina and brain provide a valuable model toward the comparison in a single organism between the conditions that contribute to the mantainance of a protein in the innate misfolded state and the conditions that contribute to the transition of a protein from an innate misfolded state toward an amyloid state in different areas of then CNS. The present results raise the question whether the physiology of the retina is altered as the presence of the PrP aggregates may interfere with the function of a synaptic transmission in a rich area like OPL. The diagnostic value of the results obtained needs to be assessed through systematic ERG and OCT studies in carriers of PRNP mutations.

### In GSS F198S, PrP deposits in the retina do not have the tinctorial properties of amyloid unlike the cores of the PrP plaques in the brain

Using thioflavin S, PrP deposits in the retina are not fluorescent whereas those in the brain are. For the detection of protein aggregates and in comparison with thioflavin S we used LCOs to determine whether retinal deposits could be detected. HS-84 has been shown to bind to Aβ and tau deposits in postmortem human brain tissue of individuals affected by AD and Pick disease, respectively[55]. pFTAA has been shown to detect PrP^SC^ aggregates in the brain of mice injected with the scrapie prion strain 22L [58]. In GSS F198S, the PrP deposits in the retina were not fluorescent using the two LCOs; however, using HS-84 and pFTAA fluorescence was present surrounding the cores of plaques in the cerebellum. Immunohistochemistry showed that Aβ immunopositivity was present surrounding the cores of many plaques suggesting that the fluorescence seen with the two LCOs is due to the presence of Aβ aggregates. In GSS A117V, the PrP deposits in the retina were not fluorescent using the two LCOs; however, the plaques in the neocortex were fluorescent with the LCOs. Previous cryo-EM studies have revealed the structure of PrP amyloid filaments extracted from the cerebellum of patients affected by GSS F198S [22]. Future cryo-EM studies of retina in GSS F198S are needed to establish whether PrP filaments can be detected at all even if the retinal inclusions do not have the tinctorial properties of amyloid. A challenge will be the availability of retinal tissue from GSS F198S. In addition, it is also important to determine whether the fold of the filaments from the Ab that aggregates around the PrP cores in the cerebellum is novel. The novel finding of fluorescence of the neocortical plaques in GSS A117V using LCOs suggests that the fold of the filaments of the amyloid in these plaques differs from that reported in GSS F198S.

Immunohistochemical analysis using nine antibodies that recognize different epitopes along the sequence of PrP has shown that the bead-like deposits in the OPL are immunopositive with each of them. In the brain, plaque cores and diffuse deposits were immunolabeled with various PrP antibodies; however, a major difference between retina and brain is that PrP antibodies directed to the N- and C-termini decorate the retinal beads, but do not decorate the cores of the PrP plaques. The latter are fluorescent with thioflavin S, while the retinal bead-like deposits are not. These findings suggest that the bead-like deposits are made of aggregates containing either full-length PrP or multiple peptides from across the entire PrP sequence. As part of understanding the pathogenesis of PrP deposits in the brain, further studies are needed to determine whether the diffuse PrP plaques and the PrP deposits that surround the core of the plaque are made of full-length PrP or large peptides containing both the N- or the C-terminus.

### The morphological and tinctorial features of PrP deposits are different between the retina and the brain

We compared the size of the bead-like deposits in the retina with that of the cores of the plaques in the frontal and cerebellar cortices. Due to the variation of thickness of the retina in its different parts, the PrP burden was not calculated. In the retina, the area of a single PrP-immunopositive bead-like aggregate occupies a surface area corresponding to 0.5 % of that of the entire PrP immunopositivity of a single plaque. When comparing a single PrP-immunopositive bead immunolabeld by 3F4, it is 11 % of the area of a core of a plaque as seen in thioflavin S preparations from sections of the frontal cortex.

In the brain, the plaque core has the tinctorial properties of amyloid in thioflavin S preparations, while the diffuse deposits do not have it. This difference may be related to the presence of the 8 kDa PrP fragment in the plaque cores and the absence of them in the diffuse deposits. Whether the diffuse deposits may, over time, acquire the tinctorial properties of amyloid cannot be established; therefore, we refer to them as non-amyloid deposits rather than preamyloid [18]. Overall, in brain, the area of PrP immunopositivity (PrP burden) is larger in the molecular layer of the cerebellar cortex (35.5 %) than in the frontal cortex (16.9 %). The area of PrP immunopositivity of the individual plaque, including both the thioflavin S fluorescent core and the non-fluorescent peripheral component are 65% larger in the molecular layer of the cerebellar cortex than in the frontal cortex. Similarly, the area of the thioflavin S fluorescent individual plaque core is on average 64% larger in the molecular layer of the cerebellar cortex than in the frontal cortex.

### In GSS F198S and A117V, PrP aggregates in the retina are detected only in the OPL

PrP^C^ is present throughout the mammalian visual system [1]. In the retina, PrP mRNA is expressed in the ganglion cell layer, inner nuclear layer and outer nuclear layer [24]. PrP^C^ is localized in OPL and IPL at presynaptic and postsynaptic levels as well as in the LGN [19, 24, 26, 37]. In human PrP proteinopathies, the retina has been studied by several investigators in cases of CJD. Reviewing data currently available, it is evident that the number of CJD cases studied to date is higher than those studied for GSS. The most significant studies of the retina in CJD have been carried out by Head et al, Takao et al, and Goodwill et al [20, 24, 25, 62]. Thus, information is available from the human retina in over 30 cases. Presence of PrP deposits has been documented in the outer and inner plexiform layers. The most numerous are cases of sporadic CJD type MM1; however, sporadic CJD MM2 and MM1+2 have been also reported. Similar distribution has been also reported in iatrogenic (MM1) and in variant CJD. The information on genetically-determined cases is limited to the *PRNP* with the V180I (MM1 and MM2) and M232R (MM1). By immunohistochemistry, deposits in the OPL appear to be consistently present, while in the IPL they are occassionally less evident. No deposits have been seen in other retinal layers. Our study of nine cases of GSS has consistently shown that only the OPL contains deposits of PrP. Birth sex, initial presenting signs and symptoms, age at onset, and length of clinical course do not appear to be associated with the presence of PrP deposits in the retina in the various types of CJD as well as in GSS F198S and A117V. There appears to be a consistent difference in the layer distribution of PrP aggregates between CJD and the two forms of GSS studied here. The significance of this difference requires further investigation. Perhaps it is premature to draw rigid conclusions, due to the limited number of cases described in the literature and those in cases of GSS. It is difficult to explain the difference in the presence in PrP deposits in CJD and GSS in view of the facts that PrP^C^ in normal conditions is localized in both IPL and OPL and that in GSS the mutant protein would be present in both the OPL and IPL. [20, 24, 25, 32, 53, 62, 65]. The absence of PrP immunopositivity in the LGN remains to be understood.

We have shown that in GSS F198S, the localization of PrP deposits in the OPL corresponds to the region in which synaptic connections occur between photoreceptor cells and other retinal cells. Whether the deposits are intra or extracellular remains to be determined and it would be possible if eyes from an affected mutation carrier could be harvested with a very short postmortem interval and be processed for transmission electron microscopy. It is noteworthy that some PrP was found among the dendrites of rod bipolar cells. It has been previously shown that the recombinant PrP with the *PRNP* F198S mutation causes PrP structural instability favoring its spontaneous aggregation [64]. Cryo-EM studies of PrP extracted from the cerebellar amyloid plaques have revealed the structure of the PrP filament core in *PRNP* F198S. Our results do not provide evidence for the existence of conditions leading to PrP filament formation in the OPL; however, future experiments would have to prove or disprove our hypothesis. If filaments were to be present, then it should be determined whether their structure would be similar to those in the brain.

### PrP 8 kDa internal fragment is constitutive in the brain, but not in the retina

Our results from immunohistochemistry do not provide evidence of the presence of amyloid within the bead-like deposits in the OPL. In the brain and the retina, the morphologies and biochemical properties of the deposits are distinct. We show for the first time, using immunoblots following 2D-PAGE obtained from brain homogenates of F198S brains, that the PrP bands migrate as distinct spots from 8 to 35 kDa with the same pI. These spots correspond to PrP^IF^ aggregates and their different molecular masses depend on the degree of aggregation of the multimeric forms, while in the retina, PrP^IF^ is represented by a single spot corresponding to an 8 kDa monomer. The model structure of the filament core extracted from the amyloid core of the PrP plaques and as shown by cryo-EM, reveals that the amino-acid sequence of the peptide that makes the core of the filaments corresponds to that of the 8 kDa internal fragment of PrP. The current results combined with structural data and previous results from the PrP sequence of amyloid cores of plaques support the view that the 8 kDa internal fragment identified in the brain of GSS F198S patients is constitutive and makes the amyloid of the core of the plaques.

### PK digestion generates de novo a 8 kDa internal fragment in the retina

Our results show that following the treatment of the retinal preparation with PK, an 8 kDa fragment can be generated experimentally by PrP proteolysis. Thus, one might speculate that the proteolysis leading to the formation of the constitutive PrP 8 kDa fragment results from the actions of an unidentified PK-like proteolytic factor that is present in the brain, but not in the retina. Taken together, our study supports the hypothesis that the PrP conformation in each anatomical area has a propensity toward specific protein cleavages, which result in amyloid-forming peptides if proteolytic conditions are present. Diversity in the properties of the neuronal and glial environments in brain and retina as well as diversity in the activity of still unknown tissue factors necessary in PrP proteolysis should be considered. It remains to be determined whether different PrP aggregation mechanisms operate between brain and retina.

### PrP in the retina is composed of full length PrP, by C1 and C2, C-terminal fragments and by a C-terminal glycosylated fragment of 25 kDa that is not detected in the brain

In the retina, PrP was characterized by PrP peptides that included the C-terminus. Cytosolic preparations (S2) obtained from brain and retina contained full length PrP as well as C2 and C1 fragments. The biochemical pattern of PrP^C^ appears to be similar in the brain and retina; however, an important difference between the brain and retina was a PrP band of 25 kDa, that was present only in S2 from the retina of controls and of individuals affected by GSS F198S.

The highly glycosylated PrP bands of 35–30 kDa were strong in GSS F198S and weak in controls supporting the view that enhanced PrP glycosylation is specific to GSS F198S. This is also consistent with results showing that PrP mutation induced a higher glycosylation in human neuroblastoma cells transfected with human F198S mutant PRNP [56, 64, 70]. The highly glycosylated PrP isoforms of 35–30 kDa and the 25 kDa band observed in sucrose gradients preparations of the retina have distinct biochemical properties, with the 35–30 kDa PrP isoforms sedimenting as large size aggregates and the 25 kDa was found only in the top fractions. Thus, abnormal PrP of the retina is made of full-length glycosylated PrP aggregates.

### PrP in the retina is composed of PrP aggregates with different sizes compare to brain and has lower seeding activity than PrP in the brain

The PrP optical density measured on an immunoblot of fraction 1 of the retina and the brain are similar suggesting that the amount of total PrP is similar. Since the distribution of PrP aggregates is different between the retina and the brain we conclude that there there is higher seeding activity in the brain. The biochemical results are, of course, likely related to PrP deposits in the brain and retina being composed of amyloid PrP and non-amyloid PrP, respectively, in the two distinct tissues [39]. Using RT-QuIC analysis of two subjects (D and E), we measured the seeding activity of PrP. In the retina, it was detectable down to a 10^−4^ dilution but was not detectable at the end-point dilution of 10^−6^. In contrast, brain tissue diluted down to 10^−6^ showed seeding activity, indicating that retina has at least 2 log_10_ SD_50_ lower seeding activity when compared to brain tissue. This difference may be explained by the lower amount of aggregated and insoluble PrP in the retina.

### Clinical Considerations

Data obtained from the previous clinical studies of the nine GSS subjects focused on the pregression of the motor and cognitive deficits. Even though patients might not have been aware of a visual deficit, it is not possible to determine whether any deficit was present at any time during the course of the disease since the neuro-ophtamological examination included the examination of ocular movement but did not include studies of visual acuity, visual fields, electroretinograms, and optical coherence tomography. It should be noted that as the illness progresses, such studies would not have been possible due to the progression of the movement disorder and cognitive symptoms. The description of the *ex vivo* pathologic changes in the CNS of multiple individuals from the same pedigree provides a stimulus for a systematic *in vivo* investigation of the pathophysiology of the retina. The fact that this study reports data on the changes in the late stage of disease does not exclude the possibility that the OPL might be involved early in the course of the illness. An early presence of PrP deposits has been reported in CJD patients with two month duration of disease.

In conclusion, the multidisciplinary studies carried out on brain and retina of GSS F198S have revealed important aspects of PrP aggregation in different areas of the CNS. Amyloid plaque formation is particularly high in the molecular layer of the cerebellar cortex where a large number of synapses occur between parallel fiber endings and spines of the Purkinje cells. The analysis of the retina has shown that a full-length highly glycosylated PrP, confined to the OPL, is not forming amyloid. This PrP is located in an area high in synapses related to the connection of photoreceptors with bipolar cells.

## Supplementary Material

1

Anamnestic Data; Supplemental Figures 1 – 6

## References

[1] Human Protein Atlas. [cited 2026 06/24/2026]; Available from: https://www.proteinatlas.org/ENSG00000171867-PRNP/tissue/retina.

[2] Björk L., Klingstedt T., and Nilsson K.P.R., Thiophene-Based Ligands: Design, Synthesis and Their Utilization for Optical Assignment of Polymorphic-Disease-Associated Protein Aggregates. Chembiochem, 2023. 24(11): p. e202300044.DOI: 10.1002/cbic.202300044. 36891883 PMC10404026

[3] Bongianni M., Orrù C., Groveman B.R., Sacchetto L., Fiorini M., Tonoli G., Triva G., Capaldi S., Testi S., Ferrari S., Cagnin A., Ladogana A., Poleggi A., Colaizzo E., Tiple D., Vaianella L., Castriciano S., Marchioni D., Hughson A.G., Imperiale D., Cattaruzza T., Fabrizi G.M., Pocchiari M., Monaco S., Caughey B., and Zanusso G., Diagnosis of Human Prion Disease Using Real-Time Quaking-Induced Conversion Testing of Olfactory Mucosa and Cerebrospinal Fluid Samples. JAMA Neurol, 2017. 74(2): p. 155–162.DOI: 10.1001/jamaneurol.2016.4614. 27942718

[4] Bugiani O., Giaccone G., Verga L., Pollo B., Frangione B., Farlow M.R., Tagliavini F., and Ghetti B., Beta PP participates in PrP-amyloid plaques of Gerstmann-Sträussler-Scheinker disease, Indiana kindred. J Neuropathol Exp Neurol, 1993. 52(1): p. 64–70.DOI: 10.1097/00005072-199301000-00008. 8093899

[5] Calvo-Rodriguez M., Hou S.S., Snyder A.C., Dujardin S., Shirani H., Nilsson K.P.R., and Bacskai B.J., In vivo detection of tau fibrils and amyloid β aggregates with luminescent conjugated oligothiophenes and multiphoton microscopy. Acta Neuropathol Commun, 2019. 7(1): p. 171.DOI: 10.1186/s40478-019-0832-1. 31703739 PMC6839235

[6] Chen S.G., Teplow D.B., Parchi P., Teller J.K., Gambetti P., and Autilio-Gambetti L., Truncated forms of the human prion protein in normal brain and in prion diseases. J Biol Chem, 1995. 270(32): p. 19173–80.DOI: 10.1074/jbc.270.32.19173. 7642585

[7] Cracco L., Xiao X., Nemani S.K., Lavrich J., Cali I., Ghetti B., Notari S., Surewicz W.K., and Gambetti P., Gerstmann-Sträussler-Scheinker disease revisited: accumulation of covalently-linked multimers of internal prion protein fragments. Acta Neuropathol Commun, 2019. 7(1): p. 85.DOI: 10.1186/s40478-019-0734-2. 31142381 PMC6540574

[8] Deters K.D., Risacher S.L., Yoder K.K., Oblak A.L., Unverzagt F.W., Murrell J.R., Epperson F., Tallman E.F., Quaid K.A., Farlow M.R., Saykin A.J., and Ghetti B., [(11)C]PiB PET in Gerstmann-Sträussler-Scheinker disease. Am J Nucl Med Mol Imaging, 2016. 6(1): p. 84–93. 27069768 PMC4749507

[9] Dlouhy S.R., Hsiao K., Farlow M.R., Foroud T., Conneally P.M., Johnson P., Prusiner S.B., Hodes M.E., and Ghetti B., Linkage of the Indiana kindred of Gerstmann-Sträussler-Scheinker disease to the prion protein gene. Nat Genet, 1992. 1(1): p. 64–7.DOI: 10.1038/ng0492-64. 1363809

[10] Europe, C.o., Additional Protocol to the Convention on Human Rights and Biomedicine, concerning Biomedical Research. CETS No. 195. Strasbourg, 25.I.2005. 2005: https://www.coe.int/en/web/conventions/full-list/-/conventions/treaty/195

[11] Farlow M.R., Yee R.D., Dlouhy S.R., Conneally P.M., Azzarelli B., and Ghetti B., Gerstmann-Sträussler-Scheinker disease. I. Extending the clinical spectrum. Neurology, 1989. 39(11): p. 1446–52.DOI: 10.1212/wnl.39.11.1446. 2812321

[12] Gerstmann J., Sträussler E., and Scheinker I., Über eine eigenartige hereditär-familiäre Erkrankung des Zentralnervensystems: zugleich ein Beitrag zur Frage des vorzeitigen lokalen Alterns. Zeitschrift für die gesamte Neurologie und Psychiatrie, 1935. 154(1): p. 736–762.

[13] Ghetti B., Tagliavini F., Masters C.L., Beyreuther K., Giaccone G., Verga L., Farlow M.R., Conneally P.M., Dlouhy S.R., Azzarelli B., and , Gerstmann-Sträussler-Scheinker disease. II. Neurofibrillary tangles and plaques with PrP-amyloid coexist in an affected family. Neurology, 1989. 39(11): p. 1453–61.DOI: 10.1212/wnl.39.11.1453. 2573006

[14] Ghetti B., Tagliavini F., Giaccone G., Bugiani O., Frangione B., Farlow M.R., and Dlouhy S.R., Familial Gerstmann-Sträussler-Scheinker disease with neurofibrillary tangles. Mol Neurobiol, 1994. 8(1): p. 41–8.DOI: 10.1007/bf02778006. 7916191

[15] Ghetti B., Dlouhy S.R., Giaccone G., Bugiani O., Frangione B., Farlow M.R., and Tagliavini F., Gerstmann-Sträussler-Scheinker disease and the Indiana kindred. Brain Pathol, 1995. 5(1): p. 61–75.DOI: 10.1111/j.1750-3639.1995.tb00578.x. 7767492

[16] Ghetti B., Piccardo P., and Zanusso G., Dominantly inherited prion protein cerebral amyloidoses - a modern view of Gerstmann-Sträussler-Scheinker. Handb Clin Neurol, 2018. 153: p. 243–269.DOI: 10.1016/b978-0-444-63945-5.00014-3. 29887140

[17] Giaccone G., Tagliavini F., Verga L., Frangione B., Farlow M.R., Bugiani O., and Ghetti B., Neurofibrillary tangles of the Indiana kindred of Gerstmann-Sträussler-Scheinker disease share antigenic determinants with those of Alzheimer disease. Brain Res, 1990. 530(2): p. 325–9.DOI: 10.1016/0006-8993(90)91304-y. 2176119

[18] Giaccone G., Verga L., Bugiani O., Frangione B., Serban D., Prusiner S.B., Farlow M.R., Ghetti B., and Tagliavini F., Prion protein preamyloid and amyloid deposits in Gerstmann-Sträussler-Scheinker disease, Indiana kindred. Proc Natl Acad Sci U S A, 1992. 89(19): p. 9349–53.DOI: 10.1073/pnas.89.19.9349. 1357663 PMC50124

[19] Gong J., Jellali A., Forster V., Mutterer J., Dubus E., Altrock W.D., Sahel J.A., Rendon A., and Picaud S., The toxicity of the PrP106–126 prion peptide on cultured photoreceptors correlates with the prion protein distribution in the mammalian and human retina. Am J Pathol, 2007. 170(4): p. 1314–24.DOI: 10.2353/ajpath.2007.060340. 17392170 PMC1829464

[20] Goodwill V.S., Dryden I., Choi J., De Lillo C., Soldau K., Llibre-Guerra J., Sanchez H., Sigurdson C.J., and Lin J.H., Minimal change prion retinopathy: Morphometric comparison of retinal and brain prion deposits in Creutzfeldt-Jakob disease. Exp Eye Res, 2022. 222: p. 109172.DOI: 10.1016/j.exer.2022.109172. 35803332 PMC9946801

[21] Hallinan G.I., Hoq M.R., Ghosh M., Vago F.S., Fernandez A., Garringer H.J., Vidal R., Jiang W., and Ghetti B., Structure of Tau filaments in Prion protein amyloidoses. Acta Neuropathol, 2021. 142(2): p. 227–241.DOI: 10.1007/s00401-021-02336-w. 34128081 PMC8270882

[22] Hallinan G.I., Ozcan K.A., Hoq M.R., Cracco L., Vago F.S., Bharath S.R., Li D., Jacobsen M., Doud E.H., Mosley A.L., Fernandez A., Garringer H.J., Jiang W., Ghetti B., and Vidal R., Cryo-EM structures of prion protein filaments from Gerstmann-Sträussler-Scheinker disease. Acta Neuropathol, 2022. 144(3): p. 509–520.DOI: 10.1007/s00401-022-02461-0. 35819518 PMC9381446

[23] Head M., Ironside J., Ghetti B., Jeffrey M., Piccardo P., and Will B., Prion Diseases, in Greenfield’s Neuropathology, Love S., Budka H., and Perry A., Editors. 2015, CRC PRESS-TAYLOR & FRANCIS GROUP: Boca Raton. p. 1016–1086.

[24] Head M.W., Northcott V., Rennison K., Ritchie D., McCardle L., Bunn T.J., McLennan N.F., Ironside J.W., Tullo A.B., and Bonshek R.E., Prion protein accumulation in eyes of patients with sporadic and variant Creutzfeldt-Jakob disease. Invest Ophthalmol Vis Sci, 2003. 44(1): p. 342–6.DOI: 10.1167/iovs.01-1273. 12506094

[25] Head M.W., Peden A.H., Yull H.M., Ritchie D.L., Bonshek R.E., Tullo A.B., and Ironside J.W., Abnormal prion protein in the retina of the most commonly occurring subtype of sporadic Creutzfeldt-Jakob disease. Br J Ophthalmol, 2005. 89(9): p. 1131–3.DOI: 10.1136/bjo.2004.063495. 16113366 PMC1772846

[26] Herms J., Tings T., Gall S., Madlung A., Giese A., Siebert H., Schürmann P., Windl O., Brose N., and Kretzschmar H., Evidence of presynaptic location and function of the prion protein. J Neurosci, 1999. 19(20): p. 8866–75.DOI: 10.1523/jneurosci.19-20-08866.1999. 10516306 PMC6782778

[27] Hsiao K., Baker H.F., Crow T.J., Poulter M., Owen F., Terwilliger J.D., Westaway D., Ott J., and Prusiner S.B., Linkage of a prion protein missense variant to Gerstmann-Sträussler syndrome. Nature, 1989. 338(6213): p. 342–5.DOI: 10.1038/338342a0. 2564168

[28] Hsiao K., Dlouhy S.R., Farlow M.R., Cass C., Da Costa M., Conneally P.M., Hodes M.E., Ghetti B., and Prusiner S.B., Mutant prion proteins in Gerstmann-Sträussler-Scheinker disease with neurofibrillary tangles. Nat Genet, 1992. 1(1): p. 68–71.DOI: 10.1038/ng0492-68. 1363810

[29] Ishikawa A., Tanikawa A., Shimada Y., Mutoh T., Yamamoto H., and Horiguchi M., Electroretinograms in three cases of Creutzfeldt-Jakob disease with visual disturbances. Jpn J Ophthalmol, 2009. 53(1): p. 31–34.DOI: 10.1007/s10384-008-0608-9. 19184306

[30] Jiménez-Huete A., Lievens P.M., Vidal R., Piccardo P., Ghetti B., Tagliavini F., Frangione B., and Prelli F., Endogenous proteolytic cleavage of normal and disease-associated isoforms of the human prion protein in neural and non-neural tissues. Am J Pathol, 1998. 153(5): p. 1561–72.DOI: 10.1016/s0002-9440(10)65744-6. 9811348 PMC1853409

[31] Kitamoto T., Ogomori K., Tateishi J., and Prusiner S.B., Formic acid pretreatment enhances immunostaining of cerebral and systemic amyloids. Lab Invest, 1987. 57(2): p. 230–6. 2441141

[32] Kitamoto T., Shin R.W., Doh-ura K., Tomokane N., Miyazono M., Muramoto T., and Tateishi J., Abnormal isoform of prion proteins accumulates in the synaptic structures of the central nervous system in patients with Creutzfeldt-Jakob disease. Am J Pathol, 1992. 140(6): p. 1285–94. 1351366 PMC1886543

[33] Klingstedt T., Aslund A., Simon R.A., Johansson L.B., Mason J.J., Nyström S., Hammarström P., and Nilsson K.P., Synthesis of a library of oligothiophenes and their utilization as fluorescent ligands for spectral assignment of protein aggregates. Org Biomol Chem, 2011. 9(24): p. 8356–70.DOI: 10.1039/c1ob05637a. 22051883 PMC3326384

[34] Kretzschmar H.A., Stowring L.E., Westaway D., Stubblebine W.H., Prusiner S.B., and Dearmond S.J., Molecular cloning of a human prion protein cDNA. DNA, 1986. 5(4): p. 315–24.DOI: 10.1089/dna.1986.5.315. 3755672

[35] London A., Benhar I., and Schwartz M., The retina as a window to the brain-from eye research to CNS disorders. Nat Rev Neurol, 2013. 9(1): p. 44–53.DOI: 10.1038/nrneurol.2012.227. 23165340

[36] Mai J.K., Majtanik M., and Paxinos G., Atlas of the Human Brain. 2015: Elsevier Science.

[37] Mironov A. Jr., Latawiec D., Wille H., Bouzamondo-Bernstein E., Legname G., Williamson R.A., Burton D., DeArmond S.J., Prusiner S.B., and Peters P.J., Cytosolic prion protein in neurons. J Neurosci, 2003. 23(18): p. 7183–93.DOI: 10.1523/jneurosci.23-18-07183.2003. 12904479 PMC6740654

[38] Monaco S., Fiorini M., Farinazzo A., Ferrari S., Gelati M., Piccardo P., Zanusso G., and Ghetti B., Allelic origin of protease-sensitive and protease-resistant prion protein isoforms in Gerstmann-Sträussler-Scheinker disease with the P102L mutation. PLoS One, 2012. 7(2): p. e32382.DOI: 10.1371/journal.pone.0032382. 22384235 PMC3285667

[39] Morales R., Hu P.P., Duran-Aniotz C., Moda F., Diaz-Espinoza R., Chen B., Bravo-Alegria J., Makarava N., Baskakov I.V., and Soto C., Strain-dependent profile of misfolded prion protein aggregates. Sci Rep, 2016. 6: p. 20526.DOI: 10.1038/srep20526. 26877167 PMC4753423

[40] Murrell J., Farlow M., Ghetti B., and Benson M.D., A mutation in the amyloid precursor protein associated with hereditary Alzheimer’s disease. Science, 1991. 254(5028): p. 97–9.DOI: 10.1126/science.1925564. 1925564

[41] Nafe R., Arendt C.T., and Hattingen E., Human prion diseases and the prion protein - what is the current state of knowledge? Transl Neurosci, 2023. 14(1): p. 20220315.DOI: 10.1515/tnsci-2022-0315. 37854584 PMC10579786

[42] Nag T.C. and Wadhwa S., Differential expression of syntaxin-1 and synaptophysin in the developing and adult human retina. J Biosci, 2001. 26(2): p. 179–91.DOI: 10.1007/bf02703642. 11426054

[43] Orrú C.D., Bongianni M., Tonoli G., Ferrari S., Hughson A.G., Groveman B.R., Fiorini M., Pocchiari M., Monaco S., Caughey B., and Zanusso G., A test for Creutzfeldt-Jakob disease using nasal brushings. N Engl J Med, 2014. 371(6): p. 519–29.DOI: 10.1056/NEJMoa1315200. 25099576 PMC4186748

[44] Orrú C.D., Groveman B.R., Raymond L.D., Hughson A.G., Nonno R., Zou W., Ghetti B., Gambetti P., and Caughey B., Bank Vole Prion Protein As an Apparently Universal Substrate for RT-QuIC-Based Detection and Discrimination of Prion Strains. PLoS Pathog, 2015. 11(6): p. e1004983.DOI: 10.1371/journal.ppat.1004983. 26086786 PMC4472236

[45] Orrù C.D., Soldau K., Cordano C., Llibre-Guerra J., Green A.J., Sanchez H., Groveman B.R., Edland S.D., Safar J.G., Lin J.H., Caughey B., Geschwind M.D., and Sigurdson C.J., Prion Seeds Distribute throughout the Eyes of Sporadic Creutzfeldt-Jakob Disease Patients. mBio, 2018. 9(6).DOI: 10.1128/mBio.02095-18. PMC6247090 30459197

[46] Piccardo P., Ghetti B., Dickson D.W., Vinters H.V., Giaccone G., Bugiani O., Tagliavini F., Young K., Dlouhy S.R., Seiler C., and , Gerstmann-Sträussler-Scheinker disease (PRNP P102L): amyloid deposits are best recognized by antibodies directed to epitopes in PrP region 90–165. J Neuropathol Exp Neurol, 1995. 54(6): p. 790–801.DOI: 10.1097/00005072-199511000-00006. 7595652

[47] Piccardo P., Seiler C., Dlouhy S.R., Young K., Farlow M.R., Prelli F., Frangione B., Bugiani O., Tagliavini F., and Ghetti B., Proteinase-K-resistant prion protein isoforms in Gerstmann-Sträussler-Scheinker disease (Indiana kindred). J Neuropathol Exp Neurol, 1996. 55(11): p. 1157–63.DOI: 10.1097/00005072-199611000-00007. 8939199

[48] Piccardo P., Liepnieks J.J., William A., Dlouhy S.R., Farlow M.R., Young K., Nochlin D., Bird T.D., Nixon R.R., Ball M.J., DeCarli C., Bugiani O., Tagliavini F., Benson M.D., and Ghetti B., Prion proteins with different conformations accumulate in Gerstmann-Sträussler-Scheinker disease caused by A117V and F198S mutations. Am J Pathol, 2001. 158(6): p. 2201–7.DOI: 10.1016/s0002-9440(10)64692-5. 11395398 PMC1891977

[49] Rasmussen J., Mahler J., Beschorner N., Kaeser S.A., Häsler L.M., Baumann F., Nyström S., Portelius E., Blennow K., Lashley T., Fox N.C., Sepulveda-Falla D., Glatzel M., Oblak A.L., Ghetti B., Nilsson K.P.R., Hammarström P., Staufenbiel M., Walker L.C., and Jucker M., Amyloid polymorphisms constitute distinct clouds of conformational variants in different etiological subtypes of Alzheimer’s disease. Proc Natl Acad Sci U S A, 2017. 114(49): p. 13018–13023.DOI: 10.1073/pnas.1713215114. 29158413 PMC5724274

[50] Richard P., Renault F., Ostré C., and Auzoux-Chevé M., Neurophysiological follow-up in two children with Creutzfeldt-Jakob disease after human growth hormone treatment. Electroencephalogr Clin Neurophysiol, 1994. 91(2): p. 100–7.DOI: 10.1016/0013-4694(94)90031-0. 7519139

[51] Risacher S.L., Farlow M.R., Bateman D.R., Epperson F., Tallman E.F., Richardson R., Murrell J.R., Unverzagt F.W., Apostolova L.G., Bonnin J.M., Ghetti B., and Saykin A.J., Detection of tau in Gerstmann-Sträussler-Scheinker disease (PRNP F198S) by [(18)F]Flortaucipir PET. Acta Neuropathol Commun, 2018. 6(1): p. 114.DOI: 10.1186/s40478-018-0608-z. 30373672 PMC6205777

[52] Sakuma T., Watanabe S., Ouchi A., Sakanishi Y., Mizota A., and Ebihara N., Three Cases of Creutzfeldt-Jakob Disease with Visual Disturbances as Initial Manifestation. Case Rep Ophthalmol, 2019. 10(3): p. 349–356.DOI: 10.1159/000503274. 31762767 PMC6873069

[53] Salès N., Rodolfo K., Hässig R., Faucheux B., Di Giamberardino L., and Moya K.L., Cellular prion protein localization in rodent and primate brain. Eur J Neurosci, 1998. 10(7): p. 2464–71.DOI: 10.1046/j.1460-9568.1998.00258.x. 9749773

[54] Schlote W., Boellaard J.W., Schumm F., and St hr M., Gerstmann-Sträussler-Scheinker’s disease. Electron-microscopic observations on a brain biopsy. Acta Neuropathol, 1980. 52(3): p. 203–11.DOI: 10.1007/bf00705808. 7445983

[55] Schweighauser M., Garringer H.J., Klingstedt T., Nilsson K.P.R., Masuda-Suzukake M., Murrell J.R., Risacher S.L., Vidal R., Scheres S.H.W., Goedert M., Ghetti B., and Newell K.L., Mutation ΔK281 in MAPT causes Pick’s disease. Acta Neuropathologica, 2023. 146(2): p. 211–226.DOI: 10.1007/s00401-023-02598-6. 37351604 PMC10329087

[56] Sevillano A.M., Aguilar-Calvo P., Kurt T.D., Lawrence J.A., Soldau K., Nam T.H., Schumann T., Pizzo D.P., Nyström S., Choudhury B., Altmeppen H., Esko J.D., Glatzel M., Nilsson K.P.R., and Sigurdson C.J., Prion protein glycans reduce intracerebral fibril formation and spongiosis in prion disease. J Clin Invest, 2020. 130(3): p. 1350–1362.DOI: 10.1172/jci131564. 31985492 PMC7269597

[57] Shirani H., Linares M., Sigurdson C.J., Lindgren M., Norman P., and Nilsson K.P., A Palette of Fluorescent Thiophene-Based Ligands for the Identification of Protein Aggregates. Chemistry, 2015. 21(43): p. 15133–7.DOI: 10.1002/chem.201502999. 26388448 PMC4641461

[58] Stepanchuk A., Tahir W., Nilsson K.P.R., Schatzl H.M., and Stys P.K., Early detection of prion protein aggregation with a fluorescent pentameric oligothiophene probe using spectral confocal microscopy. J Neurochem, 2021. 156(6): p. 1033–1048.DOI: 10.1111/jnc.15148. 32799317

[59] Striebel J.F., Carroll J.A., Race B., Leung J.M., Schwartz C., Reese E.D., Bowes Rickman C., Chesebro B., and Klingeborn M., The prion protein is required for normal responses to light stimuli by photoreceptors and bipolar cells. iScience, 2024. 27(10): p. 110954.DOI: 10.1016/j.isci.2024.110954. 39381753 PMC11460503

[60] Tagliavini F., Prelli F., Ghiso J., Bugiani O., Serban D., Prusiner S.B., Farlow M.R., Ghetti B., and Frangione B., Amyloid protein of Gerstmann-Sträussler-Scheinker disease (Indiana kindred) is an 11 kd fragment of prion protein with an N-terminal glycine at codon 58. Embo j, 1991. 10(3): p. 513–9.DOI: 10.1002/j.1460-2075.1991.tb07977.x. 1672107 PMC452678

[61] Tagliavini F., Prelli F., Porro M., Rossi G., Giaccone G., Farlow M.R., Dlouhy S.R., Ghetti B., Bugiani O., and Frangione B., Amyloid fibrils in Gerstmann-Sträussler-Scheinker disease (Indiana and Swedish kindreds) express only PrP peptides encoded by the mutant allele. Cell, 1994. 79(4): p. 695–703.DOI: 10.1016/0092-8674(94)90554-1. 7954833

[62] Takao M., Kimura H., Kitamoto T., and Mihara B., PrP(res) deposition in the retina is a common finding of sporadic, familial and iatrogenic Creutzfeldt-Jakob diseases (CJD). Acta Neuropathol Commun, 2018. 6(1): p. 78.DOI: 10.1186/s40478-018-0582-5. 30097055 PMC6086066

[63] Unverzagt F.W., Farlow M.R., Norton J., Dlouhy S.R., Young K., and Ghetti B., Neuropsychological function in patients with Gerstmann-Sträussler-Scheinker disease from the Indiana kindred (F198S). J Int Neuropsychol Soc, 1997. 3(2): p. 169–78. 9126858

[64] Vanik D.L. and Surewicz W.K., Disease-associated F198S mutation increases the propensity of the recombinant prion protein for conformational conversion to scrapie-like form. J Biol Chem, 2002. 277(50): p. 49065–70.DOI: 10.1074/jbc.M207511200. 12372829

[65] Wadsworth J.D., Joiner S., Hill A.F., Campbell T.A., Desbruslais M., Luthert P.J., and Collinge J., Tissue distribution of protease resistant prion protein in variant Creutzfeldt-Jakob disease using a highly sensitive immunoblotting assay. Lancet, 2001. 358(9277): p. 171–80.DOI: 10.1016/s0140-6736(01)05403-4. 11476832

[66] Wilham J.M., Orrú C.D., Bessen R.A., Atarashi R., Sano K., Race B., Meade-White K.D., Taubner L.M., Timmes A., and Caughey B., Rapid end-point quantitation of prion seeding activity with sensitivity comparable to bioassays. PLoS Pathog, 2010. 6(12): p. e1001217.DOI: 10.1371/journal.ppat.1001217. 21152012 PMC2996325

[67] World Medical Association, World Medical Association Declaration of Helsinki: ethical principles for medical research involving human subjects. Jama, 2013. 310(20): p. 2191–4.DOI: 10.1001/jama.2013.281053. 24141714

[68] Yamaguchi K., Cochran E.J., Murrell J.R., Polymeropoulos M.H., Shannon K.M., Crowther R.A., Goedert M., and Ghetti B., Abundant neuritic inclusions and microvacuolar changes in a case of diffuse Lewy body disease with the A53T mutation in the alpha-synuclein gene. Acta Neuropathol, 2005. 110(3): p. 298–305.DOI: 10.1007/s00401-005-1042-4. 15981014

[69] Yee R.D., Farlow M.R., Suzuki D.A., Betelak K.F., and Ghetti B., Abnormal eye movements in Gerstmann-Sträussler-Scheinker disease. Arch Ophthalmol, 1992. 110(1): p. 68–74.DOI: 10.1001/archopht.1992.01080130070028. 1731725

[70] Zaidi S.I., Richardson S.L., Capellari S., Song L., Smith M.A., Ghetti B., Sy M.S., Gambetti P., and Petersen R.B., Characterization of the F198S prion protein mutation: enhanced glycosylation and defective refolding. J Alzheimers Dis, 2005. 7(2): p. 159–71; discussion 173–80.DOI: 10.3233/jad-2005-7209. 15851854

[71] Zanusso G., Ferrari S., Cardone F., Zampieri P., Gelati M., Fiorini M., Farinazzo A., Gardiman M., Cavallaro T., Bentivoglio M., Righetti P.G., Pocchiari M., Rizzuto N., and Monaco S., Detection of pathologic prion protein in the olfactory epithelium in sporadic Creutzfeldt-Jakob disease. N Engl J Med, 2003. 348(8): p. 711–9.DOI: 10.1056/NEJMoa022043. 12594315

[72] Zanusso G., Fiorini M., Ferrari S., Meade-White K., Barbieri I., Brocchi E., Ghetti B., and Monaco S., Gerstmann-Sträussler-Scheinker disease and “anchorless prion protein” mice share prion conformational properties diverging from sporadic Creutzfeldt-Jakob disease. J Biol Chem, 2014. 289(8): p. 4870–81.DOI: 10.1074/jbc.M113.531335. 24398683 PMC3931049

