# Supplementary material for: Distinctive properties of the prion protein in the brain and retina in the amyloidosis associated with the PRNP F198S Mutation
Source: Res Sq. 2026 Jul 16:rs.3.rs-9441514. Preprint. [Version 1] doi: 10.21203/rs.3.rs-9441514/v1 (PMC13405482; doi:10.21203/rs.3.rs-9441514/v1)
Supplement: 1 [file NIHPPRS9441514V1-supplement-1.pdf]

SUPPLEMENTAL FIGURE LEGENDS

Supplemental Figure 1 - Schematic representation of human full-length prion protein and its relationship to the epitopes of the nine antibodies recognizing different regions of the protein. The signal peptides are indicated by segments 1-23 and 232-253. The internal PrP fragment is indicated by segment 80-150. The boxes correspond to the epitopes of polyclonal (vertical filled boxes) and monoclonal (solid filled boxes) antibodies used to characterize PrP deposits in the retina.

Supplemental Figure 2 - Immunofluorescent images of retinal sections from a GSS F198S affected individual. Superior calotte retinal section co-stained with 3F4 and ribbon synapse marker, Ctbp2 (a,b) from a GSS F198S affected individual. Bead-like PrP deposits (red) localized in OPL close to the ribbon synapses (green) (a,b). Magnification of boxed area in panel a (b). Co-staining of PrP and rod-bipolar cells, PKCα (c,d). Magnification of boxed area in panel c (d). White arrows indicate PrP colocalization with rod bipolar dendrites (d). Co-staining of PrP and microglia, Iba1 (e). In all panels nuclei were stained with DAPI. Bars: 100 μm (a, c, e), 10 μm (b, d). Subject: E (a-e)

Supplemental Figure 3 - Coronal sections from left cerebral hemisphere of an individual affected by GSS F198S. Coronal section (subject A) stained with LFB-H&E (a) shows a moderate cerebral atrophy and loss of the myelin stain in the subcortical white matter of superior frontal gyrus. Coronal sections (subject A) immunostained with 3F4 for PrP (b) and AT8 for tau (c) reveal immunopositivity throughout the cortex and subcortical nuclei. Bars: 5 mm (a-c). Subject: A (a-c).

Supplementary Figure 4 – Lateral geniculate nucleus from an individual affected by GSS F198S. Sections from the lateral geniculate nucleus stained with H&E (a) and Bodian silver method (b) and immunolabeled with PrP 90-108 (c). Note the lack of preservation of the layers of the lateral geniculate nucleus (a-b) and the absence of intracellular inclusions and the absence of PrP immunopositivity within the layers, but the presence of PrP immunopositivity outside the boundaries of the layers (c). Bars: 100 μm (a-c). Subject: H (a-c).

Supplemental Figure 5 – Sagittal and parasagittal sections of cerebellum from an individual affected by GSS F198S. Saggital section of the cerebellar vermis stained with thioflavin S (a) and parasagittal sections of the cerebellar hemisphere stained with LFB-H&E (b) and immunolabeled with 3F4 (c). Note the numerous fluorescent plaque cores in cerebellar cortex (a), the loss of the myelin stain in the cerebellar subcortical white matter (b) and diffuse PrP immunopositivity throughout the cerebellar cortex (c). Bars: 5 mm (a), 5 mm (b-c). Subjects: E (a), A (b-c).

Supplemental Figure 6 - Immunoblot analyses of detergent-soluble (S2) and detergent-insoluble fractions (P3) retinal and frontal cortex preparations following PNGase treatment. In S2 and P3 brain preparations, PrP migrated as a 27 kDa band while in P3 several bands of high molecular mass were seen corresponding to 8kDa PrP internal fragments (a); in the

retina, PrP showed a major band of 27 kDa and a band of 20 kDa in S2 and P3 fractions (b).Immunoblot with 6H4, in S2 brain preparation, PrP migrated as three bands of 27, 20 and 18 kDa while in P3 the first first two bands were barely detectable while C1 was clearly seen (c). In the retina preparations, in S2, C2 and C1 were seen while in P3, only the C1 fragment was detected (d).

Supplementary Files

This is a list of supplementary files associated with this preprint. Click to download.

- SupplFig1REVISIONFINAL.tif
- SupplFig2REVISIONFINAL.tif
- SupplFig3REVISIONFINAL.tif
- SupplFig4REVISIONFINAL.tif
- SupplFig5REVISIONFINAL.tif
- SupplFig6REVISIONFINAL.tif
- SubjectSupplementalMaterialREVISIONFINALJune302026.docx
